# Nanorod/nanodisk‐integrated liquid crystalline systems for starvation, chemodynamic, and photothermal therapy of cancer

**DOI:** 10.1002/btm2.10470

**Published:** 2022-12-15

**Authors:** Sungyun Kim, ChaeRim Hwang, Da In Jeong, JiHye Park, Han‐Jun Kim, KangJu Lee, Junmin Lee, Seung‐Hwan Lee, Hyun‐Jong Cho

**Affiliations:** ^1^ Department of Pharmacy College of Pharmacy, Kangwon National University Chuncheon Gangwon Republic of Korea; ^2^ Terasaki Institute for Biomedical Innovation Los Angeles California USA; ^3^ College of Pharmacy Korea University Sejong South Korea; ^4^ School of Healthcare and Biomedical Engineering Chonnam National University Yeosu Republic of Korea; ^5^ Department of Materials Science and Engineering Pohang University of Science and Technology (POSTECH) Pohang Republic of Korea; ^6^ Institute of Forest Science Kangwon National University Chuncheon Republic of Korea; ^7^ Department of Forest Biomaterials Engineering College of Forest and Environmental Sciences, Kangwon National University Chuncheon Gangwon Republic of Korea

**Keywords:** cellulose nanocrystal, chemodynamic therapy, hybrid gel network, Laponite, photothermal therapy

## Abstract

Indocyanine green (ICG), glucose oxidase (GOx), and copper(II) sulfate (Cu)‐installed hybrid gel based on organic nanorod (cellulose nanocrystal [CNC]) and inorganic nanodisk (Laponite [LAP]) was developed to perform a combination of starvation therapy (ST), chemodynamic therapy (CDT), and photothermal therapy (PTT) for localized cancers. A hybrid CNC/LAP network with a nematic phase was designed to enable instant gelation, controlled viscoelasticity, syringe injectability, and longer in vivo retention. Moreover, ICG was introduced into the CNC/LAP gel system to induce hyperthermia of tumor tissue, amplifying the CDT effect; GOx was used for glucose deprivation (related to the Warburg effect); and Cu was introduced for hydroxyl radical generation (based on Fenton‐like chemistry) and cellular glutathione (GSH) degradation in cancer cells. The ICG/GOx/Cu‐installed CNC/LAP gel in combination with near‐infrared (NIR) laser realized improved antiproliferation, cellular reactive oxygen species (ROS) generation, cellular GSH degradation, and apoptosis induction in colorectal cancer (CT‐26) cells. In addition, local injection of the CNC/ICG/GOx/Cu/LAP gel into the implanted CT‐26 tumor while irradiating it with NIR laser provided strong tumor growth suppression effects. In conclusion, the designed hybrid nanorod/nanodisk gel network can be efficiently applied to the local PTT/ST/CDT of cancer cells.

## 1 INTRODUCTION

Recently, in addition to conventional chemotherapy, various approaches such as chemodynamic therapy (CDT), immunotherapy, photodynamic therapy, photothermal therapy (PTT), and sonotherapy have been attempted with diverse pharmaceutical formulations and drug delivery systems for combating cancers.^1^, ^2^, ^3^, ^4^, ^5^, ^6^, ^7^, ^8^, ^9^, ^10^, ^11^, ^12^, ^13^, ^14^, ^15^ Among these emerging therapeutic techniques, the combination of CDT and phototherapy has been widely investigated for their reactive oxygen species (ROS)‐mediated anticancer mechanisms.^16^ Notably, CDT/PTT modalities‐installed nanocarriers have been designed, and their anticancer activities have been evaluated following their intravenous administration in animal models.^17^, ^18^ However, ensuring that the nanocarriers do not enter normal organs and tissues is impossible. Compared with the systemically administering nano‐sized delivery systems, local injection around or into the tumor tissue may alleviate the risk of such systemic toxicities. Nevertheless, the efficacy and safety of local injection formulations in cancer therapy should be thoroughly investigated.

To fabricate drug delivery systems for local injection, many synthetic and natural polymers, such as chitosan, hyaluronic acid, polyethylene glycol, poly(lactic‐co‐glycolic acid), and poloxamers, have been examined.^19^, ^20^, ^21^, ^22^, ^23^, ^24^ Among these diverse materials, cellulose nanocrystal (CNC) has exhibited significant potential in the biomedical field.^25^, ^26^ CNC is typically extracted from wood, cotton, tunicate, and bacteria, and comprises a crystalline domain processed by acid hydrolysis.^27^ CNC has a rod‐like shape with a length in the order of hundreds of nanometers and diameter of several nanometers; thus, they have a high aspect ratio (up to 70).^27^ Depending on the preparation conditions, CNC can possess negatively charged sulfate groups (–OSO_3_^−^) and carboxyl groups (–COO^−^) (above its pK_a_) or positively charged amino groups (–NH_2_) (below its pK_a_).^27^ CNC dispersions with low concentrations exhibit isotropic characteristics, whereas higher CNC concentrations induce an anisotropic nematic phase.^28^ Laponite (LAP, Na^+^_0.7_[(Si_8_Mg_5.5_Li_0.3_)O_20_(OH)_4_]^−0.7^) with a diameter of 25 nm and height of 0.92 nm has been extensively investigated for the purpose of drug delivery.^29^ Although the face of the LAP disk possesses a net negative charge, its edge exhibits a charge that is either positive or less negative than the face, depending on the adjacent pH.^29^ Thus, the combination of these two materials (CNC and LAP) can provide ideal characteristics for rheologically tuned hydrogel systems.^30^ A complex CNC/LAP network can be prepared based on electrostatic interactions and hydrogen bonding between the hydroxyl groups on the outer surfaces of these materials.^30^ Electrostatic interactions between the positively charged edges of LAP and negatively charged CNC can be induced, as shown in other examples of LAP/cellulose nanofibers and LAP/alginate.^31^, ^32^ However, although a binary mixture of CNC and LAP aimed at dye absorption was reported,^30^ it has never been practically applied to drug delivery systems.

In this study, multifunctional therapeutic modalities were entrapped in the CNC/LAP birefringent gel network, and its therapeutic potential against cancer was assessed following intratumoral injection. The following ingredients were added to realize the combined effects of PTT, ST, and CDT: indocyanine green (ICG) was introduced into the CNC/LAP network to realize PTT of localized cancer under near‐infrared (NIR) light exposure.^33^ Glucose oxidase (GOx) was used to generate H_2_O_2_ via glucose decomposition (based on the Warburg effect) to accomplish starvation therapy (ST).^34^ Copper(II) sulfate (Cu) was used to produce hydroxyl radicals by a Fenton‐like reaction (for CDT) and reduce glutathione (GSH) in cancer cells.^35^, ^36^ Although H_2_O_2_ level in cancer cell is much higher than normal cell, its insufficiency still blocks the efficient application of CDT.^37^ Therefore, spontaneous generation of H_2_O_2_ from the decomposition of intracellular glucose (by ST strategy) can elevate the production efficiency of hydroxyl radical. The combination of ST and CDT can boost the killing efficiency of cancer cells by hunger and improved radical generation. The generation of hydroxyl radical based on the “cascade effect” induced by ST/CDT and its combination with PTT in a single delivery vehicle is expected to overcome the limitations of each therapeutic strategy and provide synergistic outcomes in cancer therapy.^4^ It was reported that PTT can enhance ROS generation and GSH depletion in cancer cells.^38^ Induction of hyperthermia can accelerate ST/CDT‐based radical production efficiency in cancer treatment. Integrated strategy of ROS generation and hyperthermia induction has been widely used for enhancing anticancer efficacies.^39^, ^40^, ^41^, ^42^ Organic/inorganic hybrid nanocomposite‐based gel networks (CNC/LAP) are expected to exhibit slower biodegradation characteristics considering their individual chemical compositions.^43^, ^44^ Consequently, the improved in vivo retention of the designed nematic system between CNC and LAP in the tissue can be expected. Moreover, the sustained release of ICG/GOx/Cu can be achieved with this system. Copper chloride or ferric chloride was introduced to CNC structure for CDT of cancer and ICG was incorporated to LAP formulation for PTT somewhere.^45^, ^46^, ^47^ However, the incorporation of ICG/GOx/Cu combination to hybrid CNC/LAP system was first designed in this study, to the best of our knowledge. Intratumoral injection of the PTT/ST/CDT modality‐installed CNC/LAP gel system can provide highly efficient therapeutic treatment for cancer patients even at a lower dosing frequency.

## 2 MATERIALS AND METHODS

### 2.1 Materials

Ascorbic acid, Cu (CuSO_4_·5H_2_O), d‐glucose, fluorescein isothiocyanate (FITC), GOx (from *Aspergillus niger*), GSH, 2,2′‐biquinoline‐4,4′‐dicarboxylic acid dipotassium salt trihydrate (BCA), 2′,7′‐dichlorofluorescin diacetate (DCFH‐DA), 3,3′,5,5′‐tetramethylbenzidine dihydrochloride hydrate (TMB), 5‐sulfosalicylic acid, and 5,5′‐dithiobis (2‐nitrobenzoic acid) (DTNB) were acquired from Sigma–Aldrich (St. Louis, MO). 5,5‐Dimethyl‐1‐pyrroline N‐oxide (DMPO) and ICG were supplied by Tokyo Chemical Industry Co. Ltd. (Tokyo, Japan). CNC (lyophilized form) was purchased from Cellulose Lab (Fredericton, NB, Canada). LAP (Laponite XLG) was obtained from BYK Additives & Instruments (Wesel, Germany).

### 2.2 Investigation of interactions between CNC and LAP for gel network

The particle size properties of the CNC and LAP dispersions were investigated using light scattering and laser Doppler methods (ELS‐Z1000; Otsuka Electronics, Tokyo, Japan) according to the manufacturer's guidelines. In addition, the diameter, polydispersity index, and zeta potential values of CNC in distilled water (DW) (1 mg/ml) and LAP in DW (1.5 mg/ml) were measured.

Fourier‐transform infrared (FT‐IR) data for CNC, LAP, and CNC/LAP (50/75 weight ratio, freeze‐dried form) were obtained using a Frontier FT‐IR spectrometer (PerkinElmer Inc., Buckinghamshire, UK) and the attenuated total reflectance (ATR) sampling technique. The transmittance values of each specimen were monitored in the wavenumber range 400–4000 cm^−1^.

The crystalline/amorphous properties of CNC, LAP, and CNC/LAP (50/75 weight ratio, freeze‐dried form) were determined using X‐ray diffractometry (XRD) (D8 ADVANCE with DAVINCI, Bruker, Billerica, MA) at 40 mA and 40 kV. Diffraction angle‐dependent intensity values were detected in the 2*θ* range 10–50°. The scan step size and speed were set to 0.04° and 1 s/step, respectively.

CNC (50 mg/ml in DW), LAP (75 mg/ml in DW), and CNC/LAP (50/75 mg/ml in DW) were analyzed using a small‐angle X‐ray scattering (SAXS) spectrometer (XEUSS 2.0; Xenocs, Grenoble, France) with a wavelength of 1.542 Å. Each specimen was loaded at a point 2500 mm from the detector, and the scattering vector (*q*) range was set at 0.01–0.10 Å^−1^. The SAXS patterns were obtained using a Pilatus 300 K detector (Dectris).

CNC, LAP, and freeze‐dried CNC/LAP (50/75 mg/ml) were studied using a wide‐angle X‐ray scattering (WAXS) system (D8 DISCOVER with VANTEC500, Bruker). The scattering angle (2θ) range was 2.9–74.7° with 50 kV and 1000 μA generator conditions and a 1.542 Å wavelength (λ_CuKα_). The scattering wave vector (*q*) was calculated using the following formula: *q* = $4\pi \sin\theta /\lambda_{\mathrm{Cu}K_{\alpha }}$.^48^

The thermal properties of the freeze‐dried specimens (CNC, LAP, and CNC/LAP) were using simultaneous thermal analysis (SDT Q600, TA instruments, New Castle, DE). The heat flow and weight change data were obtained using thermogravimetric analysis (TGA). To obtain the TGA profiles of CNC, LAP, and CNC/LAP, the temperature was increased at a constant rate of 10 °C/min at 50–800 °C under a 100 ml/min N_2_ supply.

Subsequently, each specimen (2 ml) was placed in a glass vial and incubated for 6 h at room temperature. After incubation, the specimen contained in the glass vial was placed between the cross‐polarizers for polarized optical observations. The polarization properties of CNC, CNC/LAP, and CNC/ICG/GOx/Cu/LAP were analyzed using polarizing microscopy (ECLIPSE E200POL, Nikon, Tokyo, Japan). A retardation filter (λ plate) was used to enhance the optical path differences in the specimen.

The morphological shapes of CNC/LAP (1/1.5 mg/ml), CNC/LAP (50/75 mg/ml, 1:50 dilution), and CNC/ICG/GOx/Cu/LAP (50/1/0.001/1/75 mg/ml, 1:50 dilution) were characterized with transmission electron microscopy (TEM) (JEM 1010; JEOL, Tokyo, Japan). Lyophilized CNC/LAP (50/75 mg/ml) and CNC/ICG/GOx/Cu/LAP (50/1/0.001/1/75 mg/ml) were dispersed in DW at a 50‐fold dilution. Finally, each specimen was loaded onto a copper grid (with carbon film) and dried. Prior to imaging, each sample was stained with 1% uranyl acetate.

### 2.3 Fabrication of hybrid gel network and its physicochemical assessment

Gel networks were designed using a simple blending method.^49^ To prepare 1 ml of the CNC/ICG/GOx/Cu/LAP (50/1/0.001/1/75 mg/ml) gel structure, CNC (50 mg) in DW (0.3 ml) was blended with ICG/GOx/Cu dissolved in DW (0.2 ml). LAP (75 mg) in DW (0.5 ml) was then added to the CNC/ICG/GOx/Cu mixture.

The gelation features of the designed nanomaterial‐associated gel systems were investigated via inversion and injection tests.^5^, ^21^, ^23^, ^49^, ^50^ CNC (50 mg/ml), LAP (75 mg/ml), CNC/LAP (50/75 mg/ml), CNC/ICG/LAP (50/1/75 mg/ml), CNC/GOx/Cu/LAP (50/0.001/1/75 mg/ml), and CNC/ICG/GOx/Cu/LAP (50/1/0.001/1/75 mg/ml) were prepared by dispersing or dissolving each ingredient in DW and blending these parts. Aliquots (2 ml) of the prepared gel mixtures were placed in glass vials and incubated for 60 min. At 5 and 60 min, each vial was inverted to monitor its gelling nature. The injection behavior of the CNC/ICG/GOx/Cu/LAP gel was investigated by extruding it through a syringe needle. The injection image of the CNC/ICG/GOx/Cu/LAP gel (after 24 h of incubation) from the plastic syringe was taken.

The rheological features of the CNC/LAP, CNC/ICG/LAP, CNC/GOx/Cu/LAP, and CNC/ICG/GOx/Cu/LAP specimens were examined using a modular compact rheometer (MCR 302; Anton Paar GmbH, Graz, Austria) after 24 h of incubation. Strain sweep (1–402%, at 10 rad/s frequency) and frequency sweep (1–100 rad/s, at 1% strain) data were acquired at 25 °C. The shear rate‐variable shear stress and viscosity data (at 0.01–100 s^−1^ shear rate) were also obtained. To determine the liquid crystalline phase, the angular frequency (1–100 rad/s)‐dependent complex viscosity profile of CNC/ICG/GOx/Cu/LAP gel was measured.

The morphology of freeze‐dried CNC/ICG/GOx/Cu/LAP was examined using variable pressure‐field emission‐scanning electron microscopy (VP‐FE‐SEM) (SUPRA 55VP, Carl Zeiss, Oberkochen, Germany). Prior to SEM imaging, the lyophilized specimen was coated with Au under vacuum.

Atomic distribution in the freeze‐dried gel structure was investigated by SEM (JSM‐7900F, JEOL Ltd., Tokyo, Japan) linked with energy dispersive spectroscopy (EDS). Lyophilized CNC/Cu/LAP and CNC/ICG/GOx/Cu/LAP specimens were coated with Pt prior to SEM imaging.

Atomic composition of the lyophilized gel system was further studied with X‐ray photoelectron spectroscopy (XPS; K‐Alpha^+^, Thermo Fisher Scientific, East Grinstead, UK). The contents of elements in the surface of freeze‐dried CNC/Cu/LAP and CNC/ICG/GOx/Cu/LAP samples were determined by XPS.

For the extracellular GSH assay, each sample (0.5 ml) of Cu (1 mg/ml), GOx/Cu (0.001/1 mg/ml), CNC/LAP (50/75 mg/ml), and CNC/GOx/Cu/LAP (50/0.001/1/75 mg/ml) was added to a dialysis bag (molecular weight cut‐off (MWCO):12–14 kDa; Viskase Inc., Chicago, IL) and moved to a conical tube. DTNB (0.476 mg) in DW (4 ml) and/or GSH (0.740 mg) in DW (4 ml) was sequentially placed in a conical tube and incubated for 30 min at 37 °C. In case of CNC/GOx/Cu/LAP group, reaction time‐dependent absorbance profiles were acquired following incubation with the mixture of DTNB and GSH solution for 0, 60, 90, and 120 min. Each specimen was diluted with DW four times and absorbance data at 250–600 nm were obtained using an ultraviolet–visible (UV–Vis) spectrometer (Libra S80, Biochrom Ltd., Cambridge, UK).

The TMB assay was performed to detect hydroxyl radicals using a colorimetric method. Aliquots (1 ml) of GOx/Cu (0.001/1 mg/ml), CNC/LAP (50/75 mg/ml), and CNC/GOx/Cu/LAP (50/0.001/1/75 mg/ml) were individually added to the dialysis tube (MWCO:12–14 kDa; Viskase Inc.) and incubated for 24 h. Samples were then transferred to conical tubes containing _D_‐glucose (20 mM, 2.5 ml) and further incubated for 1 h. TMB (20 mM, 2.5 ml) was added to the conical tube, and the absorbance (at 650 nm) was acquired at 0, 30, and 360 min with a microplate reader (SpectraMax i3, Molecular Devices, Sunnyvale, CA).

Cascade production of hydroxyl radical from CNC/ICG/GOx/Cu/LAP gel was investigated with electron spin resonance (ESR) test. Aliquot (0.5 ml) of CNC/ICG/GOx/Cu/LAP gel was inserted to dialysis tube (MWCO: 12–14 kDa; Viskase Inc.) and that was incubated with GSH (1 mM)/d‐glucose (50 mM) solution (2.5 ml) for 30 min at 37 °C. H_2_O_2_ solution (0.06%, 2.5 ml) was added to that mixture and further incubated for 30 min at 37 °C. Then, dialyzed sample was reacted with DMPO (100 mM) at same volume ratio for 30 min prior to analysis with ESR spectrometer (JES‐X320, JEOL Ltd., Tokyo, Japan).

Aliquots (0.3 ml) of CNC/GOx/LAP (50/0.001/75 mg/ml) and CNC/GOx/Cu/LAP (50/0.001/1/75 mg/ml) were added to a dialysis tube (MWCO:12–14 kDa) and transferred to a conical tube. DW (2 ml) or d‐glucose (100 mM) in DW (2 ml) was added to a specimen‐loaded dialysis bag and incubated at 37 °C for 6 h. TiOSO_4_ in DW (30 mM, 2 ml) was then placed into the tube and further incubated for 30 min. The absorbance values (at 405 nm) of the dialyzed samples were measured using a microplate reader (SpectraMax i3, Molecular Devices).

The release profile of Cu from the fabricated gel network was tested in phosphate buffered saline (PBS, pH 6.8). Cu was analyzed using a modified BCA method as previously reported.^51^ The tested or standard sample (0.25 ml) was mixed with ascorbic acid solution (16 mg/ml, 0.05 ml), BCA solution (20 mg/ml, 0.5 ml), and DW (0.2 ml). After incubation for 30 min, the absorbance (at 563 nm) was measured using a microplate reader (SpectraMax i3, Molecular Devices). Aliquot (1 ml) of CNC/ICG/GOx/Cu/LAP gel was placed in 5 ml of PBS (pH 6.8), and a portion (0.3 ml) of release media was collected at 6, 24, 48, 72, 144, 216, 336, 504, and 672 h. The amount of Cu released was analyzed using the above method.

ICG release from the gel system and its uptake into cancer cells were evaluated using near‐infrared fluorescence (NIRF) imaging. CT‐26 cells (Korean Cell Line Bank, Seoul, Republic of Korea) were cultured in DMEM containing penicillin–streptomycin (1%, v/v) and FBS (10%, v/v). Cells were seeded in 12‐well plates (2.0 × 10^5^ cells/well) and incubated for 1 day. The determined volume (0.2 ml) of CNC/ICG/GOx/Cu/LAP (5/0.1/0.0001/0.1/7.5 mg/mL) gel was added to the Transwell insert (polyester membrane, 0.4 μm pore size; Corning Inc., Corning, NY) and transferred to a 12‐well plate. Intracellular fluorescence intensity values were measured at 0, 10, 30, 60, 120, 360, and 1440 min using VISQUE InVivo Smart (Vieworks Co., Ltd., Anyang, Republic of Korea).

The GOx release pattern of the gel system was also investigated. The fluorescence intensity of the FITC‐labeled GOx was used to detect released GOx from the gel structure. FITC (1 mg) in dimethyl sulfoxide (DMSO; 0.5 ml) and GOx (10 mg) in 0.1 M carbonate buffer (pH 9) were blended and the mixture was incubated for 4 h at room temperature in the dark. It was then transferred to a dialysis tube (MWCO: 12–14 kDa) and dialyzed against PBS (0.01 M, pH 7.4) for 1 day. The dialyzed product was then lyophilized for subsequent use. An aliquot (1 ml) of the CNC/ICG/FITC‐GOx/Cu/LAP gel (including 1 μg of FITC) was added to the conical tube, and PBS (pH 6.8, 5 ml) was used as the release medium. At determined time points (6, 24, 48, and 120 h), the medium (0.2 ml) was collected and the fluorescence intensity values were measured using a microplate reader (SpectraMax i3, Molecular Devices) at 495 nm (excitation) and 550 nm (emission).

### 2.4 Cellular anticancer potential assays

The antiproliferation potentials of ICG (with or without NIR light), GOx, Cu, CNC/LAP, CNC/ICG/LAP, CNC/GOx/Cu/LAP, CNC/ICG/GOx/LAP, CNC/ICG/Cu/LAP, and CNC/ICG/GOx/Cu/LAP (in the presence or absence of NIR light) were tested in CT‐26 cells using a colorimetric assay.^5^, ^23^ Cells were incubated for 24 h in a 96‐well plate after seeding at 5.0 × 10^3^ cells/well. ICG (5, 10, 25, 50, and 100 μg/ml), GOx (1, 5, 10, 25, and 50 ng/ml), Cu (5, 10, 25, 50, and 100 μg/ml), CNC/LAP (100/150, 250/375, 500/750, and 1000/1500 μg/ml), CNC/ICG/LAP (100/2/150, 250/5/375, 500/10/750, and 1000/20/1500 μg/ml), CNC/GOx/Cu/LAP (100/0.002/2/150, 250/0.005/5/375, 500/0.01/10/750, and 1000/0.02/20/1500 μg/ml), CNC/ICG/GOx/LAP (100/2/0.002/150, 250/5/0.005/375, 500/10/0.01/750, and 1000/20/0.02/1500 μg/ml), CNC/ICG/Cu/LAP (100/2/2/150, 250/5/5/375, 500/10/10/750, and 1000/20/20/1500 μg/ml), and CNC/ICG/GOx/Cu/LAP (100/2/0.002/2/150, 250/5/0.005/5/375, 500/10/0.01/10/750, and 1000/20/0.02/20/1500 μg/ml) were treated to the cells and NIR light (808 nm, 1 W/cm^2^, and 3 min) was applied to corresponding specimens. Subsequently, the samples were incubated with each specimen for 72 h. After removing each specimen, the cells were treated with CellTiter 96 AQ_ueous_ One Solution Cell Proliferation Assay Reagent (Promega Corp., Fitchburg, WI), and absorbance values (at 490 nm) were read using a microplate reader (SpectraMax i3, Molecular Devices).

Cellular ROS levels in the designed gel structures were analyzed using ROS Detection Reagents (Molecular Probes, Inc., Eugene, OR). CT‐26 cells were incubated for 24 h after seeding at 5.0 × 10^5^ cell/well in the 6‐well plate. Aliquots (1 ml) of CNC/LAP (1000/1500 μg/ml), GOx/Cu (0.02/20 μg/ml), ICG (20 μg/ml), ICG/GOx/Cu (20/0.02/20 μg/ml), CNC/ICG/LAP (1000/20/1500 μg/ml), CNC/GOx/Cu/LAP (1000/0.02/20/1500 μg/ml), CNC/ICG/GOx/LAP (1000/20/0.02/1500 μg/ml), CNC/ICG/Cu/LAP (1000/20/20/1500 μg/ml), and CNC/ICG/GOx/Cu/LAP (1000/20/0.02/20/1500 μg/ml) were then added to the cells. NIR light (808 nm, 1 W/cm^2^, and 5 min) was applied to each well after 6 h of incubation, and the cells were further incubated for 18 h. After removing the specimen, DCFH‐DA (10 μM) was added to the cells and incubated for 20 min at 37 °C. Cell pellets were obtained by centrifugation and the cellular fluorescence signal was detected using a flow cytometer (BD Bioscience, San Diego, CA).

The intracellular GSH levels were determined using a colorimetric assay. CT‐26 cells were incubated for 24 h after seeding in 6 wells at 5.0 × 10^5^ cells/well. Aliquots (1 ml) of Cu (20 μg/ml), CNC/LAP (1000/1500 μg/ml), GOx/Cu (0.02/20 μg/ml), ICG (20 μg/ml), ICG/GOx/Cu (20/0.02/20 μg/ml), CNC/ICG/LAP (1000/20/1500 μg/ml), CNC/GOx/Cu/LAP (1000/0.02/20/1500 μg/ml), CNC/ICG/GOx/LAP (1000/20/0.02/1500 μg/ml), CNC/ICG/Cu/LAP (1000/20/20/1500 μg/ml), and CNC/ICG/GOx/Cu/LAP (1000/20/0.02/20/1500 μg/ml) were added to the cells and further incubated for 24 h. The CNC/ICG/GOx/Cu/LAP gel‐treated cells were placed under NIR light (808 nm, 1 W/cm^2^, and 5 min). After eliminating each specimen, 5‐sulfosalicylic acid (5% w/v) was added to the cells. The freeze–thaw cycle was repeated three times to obtain cell lysates. Then, a Glutathione Colorimetric Detection Kit (Thermo Fisher Scientific, Waltham, MA) was used to measure intracellular GSH levels according to the guideline provided by the manufacturer.

ATP assay was conducted to verify the activity of GOx included in the gel system. CT‐26 cells were seeded onto 12‐well plate at 5.0 × 10^5^ cells/well and incubated for 24 h. GOx (0.01 μg/ml), CNC/LAP (500/750 μg/ml), and CNC/ICG/GOx/Cu/LAP (500/10/0.01/10/750 μg/ml) were applied to the cells and they were incubated for 24 h. CNC/ICG/GOx/Cu/LAP‐treated cells were placed under NIR laser (808 nm, 1 W/cm^2^, and 3 min). After removing the samples, cells were rinsed with cold PBS and they were treated with the reagents of ATP assay kit (Abcam, Cambridge, UK) according to the manufacturer's protocol. The absorbance of each sample was measured and ATP concentration was calculated. The relative ratio of each group to control group was plotted.

The apoptosis induction efficacy of the fabricated gel system was investigated in CT‐26 cells. Cells were incubated for 24 h after seeding in 24‐well plate at 5.0 × 10^5^ cells/well. Aliquots (0.5 ml) of CNC/LAP (500/750 μg/ml), GOx/Cu (0.01/10 μg/ml), ICG (10 μg/ml), ICG/GOx/Cu (10/0.01/10 μg/ml), CNC/ICG/LAP (500/10/750 μg/ml), CNC/GOx/Cu/LAP (500/0.01/10/750 μg/ml), CNC/ICG/GOx/LAP (500/10/0.01/750 μg/ml), CNC/ICG/Cu/LAP (500/10/10/750 μg/ml), and CNC/ICG/GOx/Cu/LAP (500/10/0.01/10/750 μg/ml) in the presence or absence of NIR light (808 nm, 1 W/cm^2^, and 5 min) were added to the cells and incubated for 12 h. All specimens were removed, and the cells were washed with PBS. Cells were pelleted by centrifugation and suspended in the FITC Annexin V Apoptosis Detection Kit (BD Pharmingen, BD Biosciences, San Jose, CA). Following co‐staining with annexin V‐FITC and propidium iodide (PI), cellular fluorescence intensity values were determined using a flow cytometer (BD Bioscience).

### 2.5 Photothermal conversion studies

In vitro photothermal properties of the gel network were assessed by monitoring temperature change patterns following NIR light exposure.^5^ Specimens of ICG (1 mg/ml), CNC/LAP (50/75 mg/ml), CNC/GOx/Cu/LAP (50/0.001/1/75 mg/ml), and CNC/ICG/GOx/Cu/LAP (50/1/0.001/1/75 mg/ml) were placed in disposable cuvettes, and NIR light (808 nm, 1 W/cm^2^, and 5 min) was irradiated on the prepared specimens. The temperature distribution was scanned at 0, 1, and 5 min using a thermal camera (FLIR E8 camera; FLIR Systems Inc., Wilsonville, OR).

The photothermal properties of the fabricated gel structures were tested in mice following subcutaneous injection.^5^ Each specimen (at 5 ml/kg) of ICG (1 mg/ml), CNC/LAP (50/75 mg/ml), CNC/GOx/Cu/LAP (50/0.001/1/75 mg/ml), and CNC/ICG/GOx/Cu/LAP (50/1/0.001/1/75 mg/ml) was subcutaneously injected into the back of the Institute of Cancer Research (ICR) mouse (male, 4 weeks old, ~20 g; Koatek, Pyeongtaek, Republic of Korea). All animal experiments were approved by the Animal Care and Use Committee of Kangwon National University. All animal studies were performed according to the National Institutes of Health Guide for the Care and Use of Laboratory Animals (NIH Publications No. 8023, revised 1978). The gel‐injected skin was exposed to NIR light (808 nm, 1 W/cm^2^, and 3 min), and whole‐body images were captured using a thermal camera on day 0 and 14.

### 2.6 Biodegradation features of gel network

The biodegradation features of the associated gel networks were assessed in mice following subcutaneous injection.^21^ Aliquots (at 5 ml/kg) of ICG/GOx/Cu (1/0.001/1 mg/ml), CNC/LAP (50/75 mg/ml), CNC/ICG/GOx/Cu (50/1/0.001/1 mg/ml), ICG/GOx/Cu/LAP (1/0.001/1/75 mg/ml), and CNC/ICG/GOx/Cu/LAP (50/1/0.001/1/75 mg/ml) were injected to the shaved dorsal part of ICR mouse subcutaneously. The remaining gel was excised from the body on day 14 and its weight was measured.

### 2.7 Systemic toxicity tests

The toxicity of the associated gel structures was examined in mice by blood chemistry tests and histological staining of vital organs.^4^, ^21^, ^23^ CNC (50 mg/ml), LAP (75 mg/ml), CNC/LAP (50/75 mg/ml), ICG/GOx/Cu (1/0.001/1 mg/ml) with NIR light, CNC/GOx/Cu/LAP (50/0.001/1/75 mg/ml), and CNC/ICG/GOx/Cu/LAP (50/1/0.001/1/75 mg/ml) with or without NIR light were injected into the dorsal skin of ICR mouse subcutaneously (at 5 ml/kg dose). The gel‐injection site was exposed to NIR light (808 nm and 1 W/cm^2^) for 3 min. Blood samples were obtained from the heart on day 14, and heparinized blood samples were collected by centrifugation for 5 min at 16,100 *g*. Serum (~0.2 ml) was analyzed to determine albumin, alanine transaminase (ALT), aspartate transaminase (AST), and blood urea nitrogen (BUN) concentrations using a Cobas 8000 C702 chemical autoanalyzer (Roche Diagnostics, Manheim, Germany).^21^, ^23^ Several vital organs (i.e., heart, kidney, liver, lung, and spleen) of the mice were isolated and immersed in formaldehyde solution (4%, v/v) for fixation prior to hematoxylin and eosin (H&E) staining. All tissue specimens were processed to form paraffin blocks and sliced into thin sections. They were then stained with H&E reagent, and images were obtained using an optical microscope (Eclipse Ts2, Nikon, Tokyo, Japan).

### 2.8 Anticancer potential tests

The local anticancer efficacy of the associated gel structures was examined in CT‐26 tumor‐implanted mice. CT‐26 cell suspension (2.0 × 10^6^ cells in 0.1 ml) was inoculated into the back of BALB/c mice (male, 4 weeks old, ~20 g; Koatek). Tumor size was estimated using the following formula: *V* (mm^3^) = (4π/3) × (longest diameter/2) × (shortest diameter/2) × (height/2).^52^ Once the average tumor volume exceeded 300 mm^3^, mice were randomly divided into six groups (control, ICG + NIR, GOx/Cu, ICG/GOx/Cu + NIR, CNC/LAP, and CNC/ICG/GOx/Cu/LAP + NIR). Each sample was injected intratumorally on day 0 and 6, at a dose of 5 ml/kg. The tumor size and body weight of the mice were measured on day 0, 2, 6, 8, and 9. NIR light (808 nm and 1 W/cm^2^) was focused on the gel‐injected region for 3 min on day 0, 2, 3, 6, and 8. The temperature of the gel‐injection site was scanned using a thermal camera during the NIR light application process (for 3 min). The excised tumor mass was weighed on day 9 and fixed in a formaldehyde solution (4%, v/v) for histological assays. The fixed specimens were embedded in paraffin blocks and sectioned into thin slices. The cells were then stained with H&E and terminal deoxynucleotidyl transferase dUTP nick‐end labeling (TUNEL) reagents.^5^, ^23^ 3,3′‐Diaminobenzidine (DAB) was selected as the chromogenic horseradish peroxidase substrate in the TUNEL assay.

### 2.9 Data analysis

All data are presented as mean ± SD. Each experiment was repeated at least thrice. Statistical analyses were performed using a two‐tailed *t*‐test and analysis of variance (ANOVA).

## 3 RESULTS AND DISCUSSION

### 3.1 Preparation of hybrid gel networks and their physicochemical characterization

**FIGURE 1:**
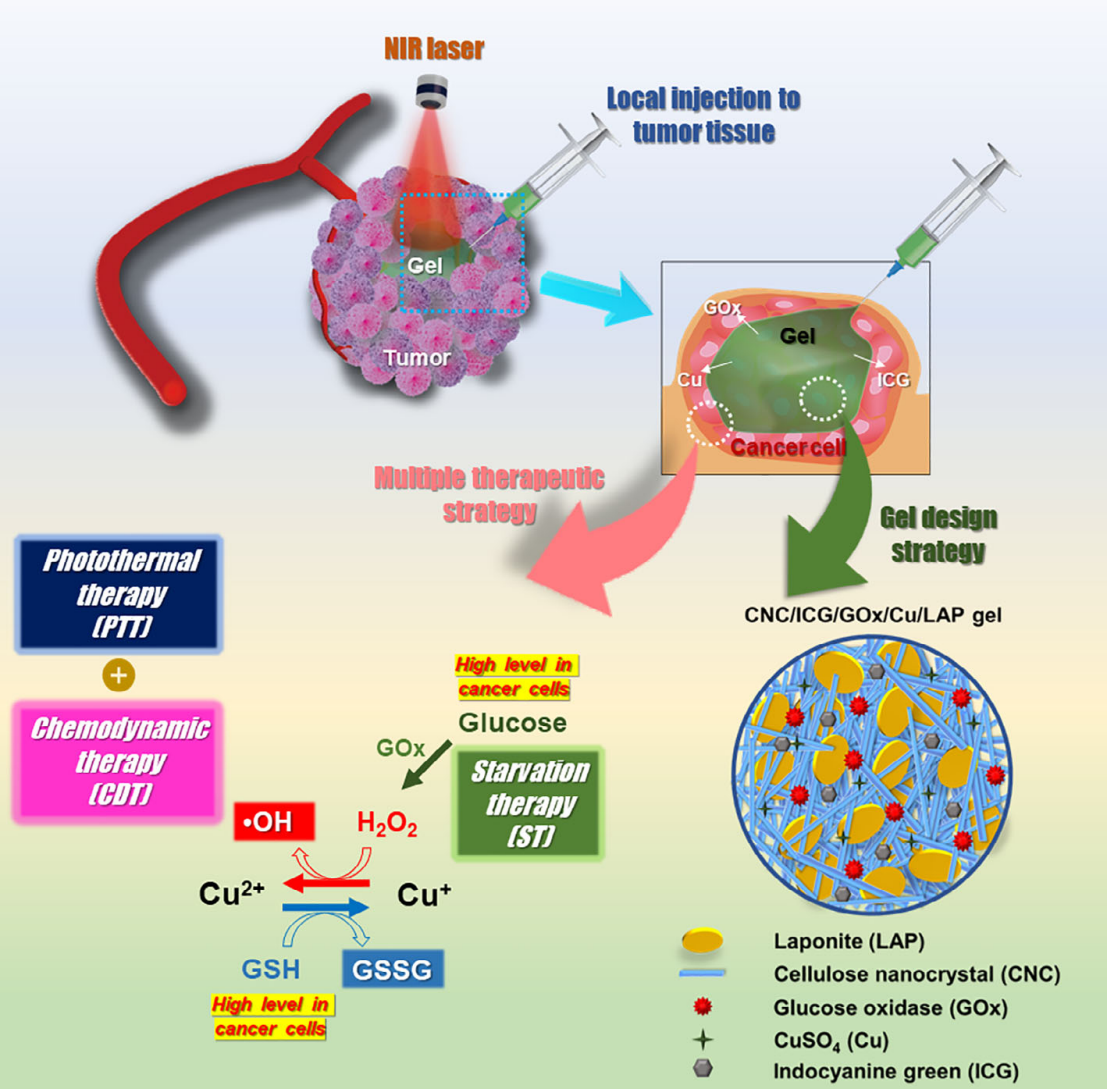
Schematic of nano‐aggregated gel network for multiple local cancer therapy.

In this study, liquid crystalline‐based gel network systems aligned with a nanorod (CNC) and nanodisk (LAP) were developed for local cancer therapy (Figure 1). Nanostructure‐based birefringent gel networks, including ICG, GOx, and Cu, were designed to realize PTT, ST, and CDT of localized cancers. Single syringe injection, immediate gelation, shear thinning behavior, and longer in vivo retention were the primary objectives of the gel network design in the current therapeutic approach. Each of the ingredients used to produce the gel network system had a specific property that could be integrated. First, GOx can catalyze the accumulation of glucose in cancer cells to H_2_O_2_ and Cu^+^ ions can produce hydroxyl radicals from H_2_O_2_ (by Fenton‐like reaction), as previously reported.^53^ Second, NIR laser absorption can potentially amplify the production of intracellular ROS, leading to enhanced anticancer activity.^53^ Finally, the PTT/ST/CDT‐enabled nanocomponent‐based gel network can be expected to exhibit combinatory anticancer effects against colon cancer via local injection.

The particle characteristics (i.e., particle size, polydispersity index, and zeta potential) of the CNC and LAP (dispersion in DW) were studied, as shown in Figures S1 and S2. The hydrodynamic diameters of the CNC and LAP dispersions, measured using the light scattering method at current concentrations, were 147 and 165 nm, respectively. Furthermore, the mean polydispersity index values of CNC and LAP dispersions were 0.24 and 0.17 with a unimodal particle size distribution (Figures S1 and S2). The zeta potential data of CNC and LAP dispersion were −44.9 and −45.7 mV, respectively (Figure S1). The LAP crystal is disk‐shaped with a diameter of 25 nm diameter and height of 0.92 nm and its empirical formula is Na^+^_0.7_[(Si_8_Mg_5.5_Li_0.3_)O_20_(OH)_4_]^−0.7^.^29^ The cation substitution between Li^+^ and Mg^2+^ induces a net negative charge on the face of LAP, which can be balanced by the positive charge of sodium ions.^29^ However, the edge of the LAP crystal may have a positive (or less negative) charge owing to the protonation of the exposed hydroxyl groups.^29^ In contrast, CNC has a rod‐like structure (with a high aspect ratio), and its surface exhibits an anionic charge owing to the presence of hydroxyl and sulfate groups.^27^, ^54^ Properties related to the particle size and surface charge were also observed in this study, and a liquid crystalline‐based gel network was fabricated by controlling the concentrations of the ingredients (CNC and LAP).

**FIGURE 2:**
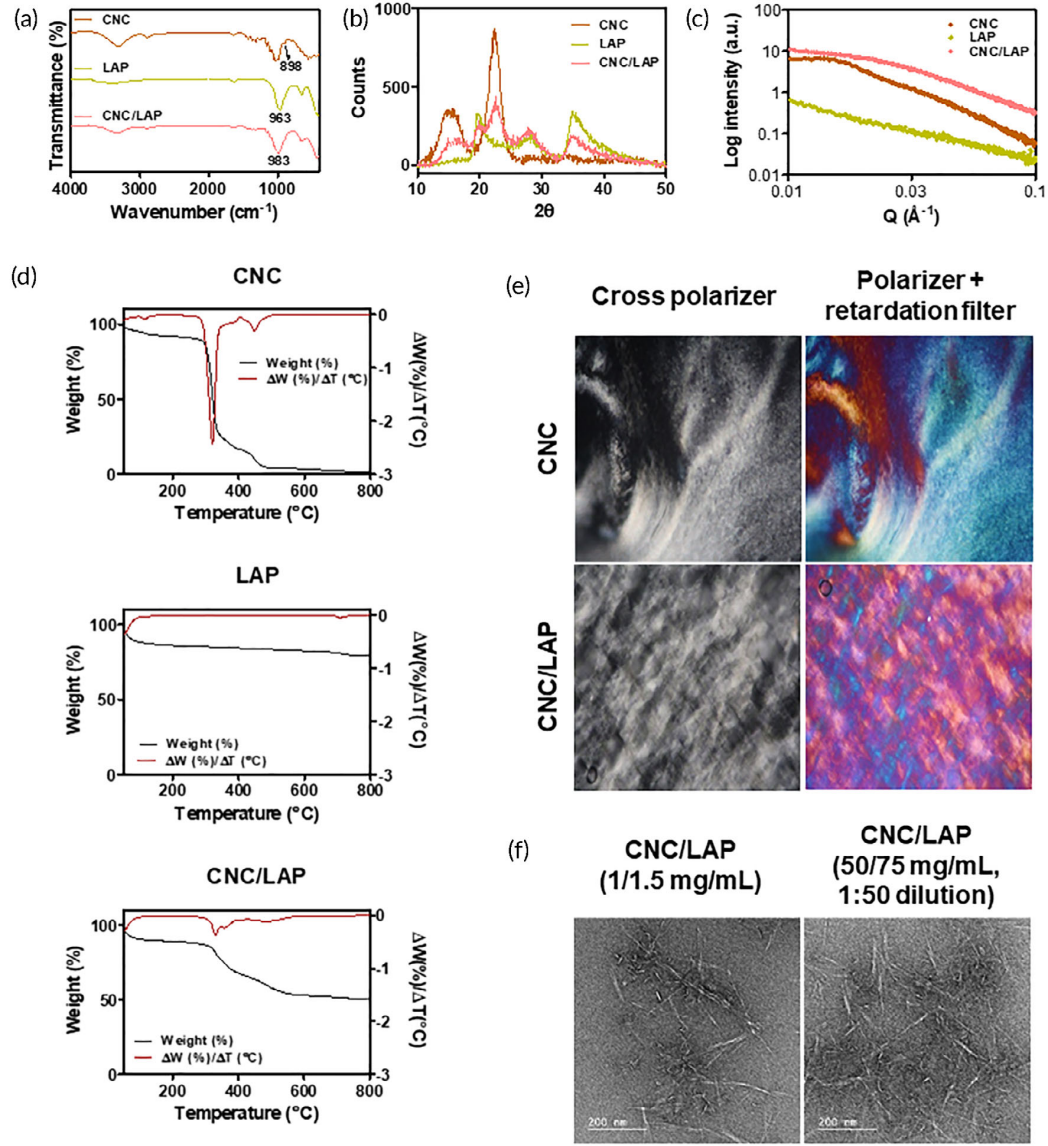
Investigation of interactions between CNC and LAP. (a) FT‐IR analysis of CNC, LAP, and CNC/LAP. Wavenumber‐dependent transmittance (%) values are plotted. (b) XRD test of CNC, LAP, and CNC/LAP. Diffraction angle (2θ)‐dependent intensity values are shown. (c) SAXS test of CNC, LAP, and CNC/LAP. Scattering vector (Q)‐dependent intensity values are plotted. (d) TGA test of CNC, LAP, and CNC/LAP. Temperature‐dependent weight change and Δ*W*/Δ*T* profiles are shown. (e) Polarizing microscopy test of CNC and CNC/LAP. (f) TEM analysis of CNC/LAP (1/1.5 mg/ml) and CNC/LAP (50/75 mg/ml, 1:50 dilution). Scale bar = 200 nm.

The interactions between CNC and LAP were investigated using FT‐IR spectroscopy (Figure 2a). In the spectrum of CNC, C–O–C stretching vibrations of (1 → 4) β‐glycosidic bonds were observed at 898 cm^−1^.^55^ The strong band at 963 cm^−1^ in the spectrum of LAP might indicate Si–O stretching, which was marginally shifted to 983 cm^−1^ in the spectrum of CNC/LAP (freeze‐dried form),^56^ indicating the interaction between CNC and LAP.

The crystalline and amorphous states of the CNC/LAP constructs were studied using XRD (Figure 2b). Diffraction peaks were observed at 15.2° and 22.4° in the profile of the CNC group, as reported in other studies.^57^, ^58^ Similarly, representative peaks (at 19.6°, 27.9°, and 34.9°) were observed in the XRD data of LAP, as previously reported.^59^ A few of the representative peaks in the CNC and LAP were also observed in the profile of the CNC/LAP group (especially at 22.6° and 34.7°). Although the interactions between CNC and LAP affect the crystalline properties, certain portions remained even after the CNC/LAP structure was formed.

The structural information of the CNC/LAP mixture was acquired using SAXS (Figure 2c). The CNC, LAP, and CNC/LAP groups exhibited a decreasing pattern in the scattering vector modulus (I(Q)) in the tested Q range. Notably, in 0.05–0.10 Å^−1^ of Q range, the exponent of the power law in CNC/LAP group was 2.2, implying the presence of gel‐like characteristics.^60^ The flat profile in the low‐Q region indicates the form factor originating from the nanorod shape of the CNC.^30^ Furthermore, the interactions between CNC and LAP were investigated using the WAXS analysis (Figure S3). The CNC group exhibited three major peaks at 1.06, 1.16, and 1.60 Å^−1^, corresponding to the (1$\bar{1}$0), (110), and (200) planes of cellulose *I*_β_.^30^ The LAP group had 001 Bragg reflections, and the CNC/LAP group also exhibited an attenuated CNC pattern and the presence of LAP peaks.^30^

The thermal properties of the fabricated CNC/LAP constructs were investigated using TGA (Figure 2d). In the CNC group, a weight loss of 5.1% was detected in the range of 30–100 °C, which can be attributed to the effect of the evaporation of water and volatile substances.^61^ The CNC group displayed a dramatic change in the degree of weight around 320 °C, as reported previously.^61^ In contrast, LAP was stable during the temperature elevation process, as reported; it merely exhibited a 20.6% weight change up to 800 °C.^55^ Furthermore, the CNC/LAP group had an intermediate feature between CNC and LAP. It exhibited a sudden mass change at approximately 330 °C; however, 50.6% of its initial mass remained at 800 °C.

The arrangement of each ingredient in the CNC/LAP structure was observed using polarization microscopy (Figure 2e). A retardation filter (λ‐plate at 530 nm) was inserted into the structure at 45° for the polarizer, and images of the CNC and CNC/LAP samples were acquired.^30^ Blue and red indicate the directions perpendicular and parallel to the slow axis of the λ‐plate in the nematic region, respectively.^30^ The birefringent feature, probably owing to the nematic phase, seemed to appear in the CNC/LAP group, considering its concentration ratio.^30^ Furthermore, in the case of the CNC/ICG/GOx/Cu/LAP group (Figure S4), an optical feature indicating the nematic phase of CNC/LAP was observed.

The morphology of the CNC/LAP dispersion was observed using TEM (Figure 2f). To ensure convenient imaging analysis, specimens with low concentrations were prepared. Both CNC/LAP (1/1.5 mg/ml) and CNC/LAP (1:50 dilution of 50/75 mg/ml) groups displayed rod‐like CNC and disk‐shaped LAP structures. In addition, the CNC/ICG/GOx/Cu/LAP group had a morphological shape similar to that of the CNC/LAP group (Figure S5).

**FIGURE 3:**
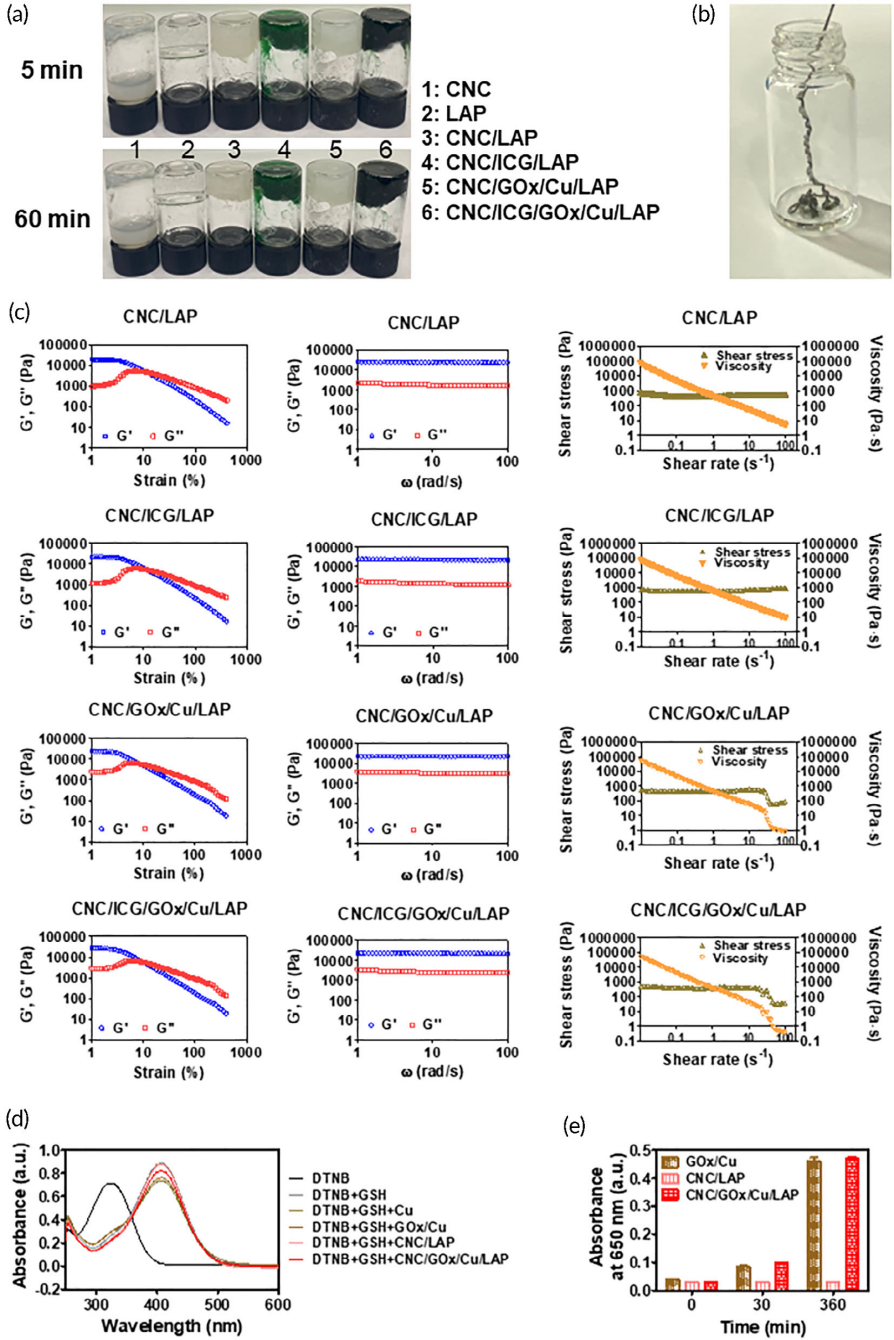
Rheological and catalytic features of CNC/LAP‐based gel systems. (a) Inversion test of CNC, LAP, CNC/LAP, CNC/ICG/LAP, CNC/GOx/Cu/LAP, and CNC/ICG/GOx/Cu/LAP groups. Images of inverted vials including each specimen after 5 and 60 min of incubation are shown. (b) Injection capability test of CNC/ICG/GOx/Cu/LAP gel. Image of injection through the syringe needle is displayed. (c) Rheology test of CNC/LAP, CNC/ICG/LAP, CNC/GOx/Cu/LAP, and CNC/ICG/GOx/Cu/LAP groups. Strain sweep, frequency sweep, and shear rate–shear stress (viscosity) data are presented. (d) Extracellular GSH assay of Cu, GOx/Cu, CNC/LAP, and CNC/GOx/Cu/LAP. Wavelength‐dependent absorbance values are displayed. (e) TMB assay of GOx/Cu, CNC/LAP, and CNC/GOx/Cu/LAP. Absorbance values at 0, 30, and 360 min are plotted. Each point represents mean ± SD (*n* = 3).

The gelation behavior of the prepared hybrid systems was evaluated using an inversion test (Figure 3a). The CNC group displayed a free‐flowing property in the inverse position, whereas the LAP group exhibited a tendency to retain its shape owing to its gel‐like behavior. Moreover, the CNC/LAP group exhibited gel‐like features; thus, it did not slip down. The incorporation of ICG and/or GOx/Cu did not disrupt the gel‐like behavior of the CNC/LAP structure, as shown in the CNC/ICG/LAP, CNC/GOx/Cu/LAP, and CNC/ICG/GOx/Cu/LAP groups. Furthermore, the possibility of performing the injection through a single syringe was studied, as shown in Figure 3b. The CNC/ICG/GOx/Cu/LAP composite could be conveniently extruded through a syringe needle despite its gel‐like nature, probably owing to its shear‐thinning feature. The observed injection capability implies that extravascular injection can easily be performed for local cancer therapy.

The viscoelastic features of the nanocomponent mixtures were investigated using a rheometer (Figure 3c and S6). In the strain sweep data, the CNC/LAP, CNC/ICG/LAP, CNC/GOx/Cu/LAP, and CNC/ICG/GOx/Cu/LAP groups exhibited a G′ > G″ pattern (indicating a gel‐like property) under 10.1%, 11.3%, 8.0%, and 10.1% strain, respectively. In addition, the G′ > G″ profile was observed in the frequency sweep data of all experimental groups, implying gel‐like properties of the mixtures. Furthermore, the shear rate–shear stress relationship was analyzed, and the shear rate‐dependent viscosity profile was acquired. A lower viscosity in the higher shear rate region indicates shear‐thinning behavior of the CNC/LAP‐based mixtures. All specimens had shear‐thinning features, which might be helpful for convenient injection through a single syringe, as shown in Figure 3b. Moreover, the addition of ICG, GOx, and Cu did not hamper the shear‐thinning properties of the CNC/LAP‐based system. The presence of a liquid crystalline phase was also demonstrated by the violation of the Cox‐Merz rule.^48^, ^62^ Finally, the frequency‐dependent complex viscosity and shear rate‐related shear viscosity profiles of CNC/ICG/GOx/Cu/LAP were compared (Figures 3c and S6). Their discordance (shear viscosity < complex viscosity in this study) denotes the breakup of the previous system owing to the presence of liquid crystalline domains.^62^

The cross‐sectional structure of the lyophilized CNC/ICG/GOx/Cu/LAP mixture was visualized using FE‐SEM (Figure S7). A similar honeycomb‐shaped structure with multiple pores was observed in the CNC/ICG/GOx/Cu/LAP mixture. This skeleton may indicate a gel‐like state following dispersion in an aqueous medium.

Elemental distribution in the lyophilized gel structure was studied by SEM with EDS mapping (Figure S8). In particular, the contents of copper in CNC/Cu/LAP and CNC/ICG/GOx/Cu/LAP gel systems were determined. Weight values of copper in CNC/Cu/LAP and CNC/ICG/GOx/Cu/LAP groups were 0.53% and 0.33%, respectively. They were similar with the calculated weight ratios of copper in freeze‐dried gel samples. Although the weight percentage of copper was lower than the other elements, its distribution in the lyophilized gel specimen was also seen in the image. The atom contents in the surface of freeze‐dried gel samples were also measured by XPS analysis (Figure S9). The contents of Cu 2p in both CNC/Cu/LAP and CNC/ICG/GOx/Cu/LAP groups were lower than the other elements, however its existence in the surface of lyophilized specimen was identified.

The effect of copper ions on GSH depletion was investigated using an extracellular GSH assay (Figures 3d and S10). Cu^2+^ ions may convert GSH to GSSG and GSH can transform DTNB to 2‐nitro‐5‐thiobenzoic acid (TNB).^63^ The DTNB and DTNB + GSH groups had maximum peaks at 321 and 409 nm, respectively, as shown in our previous study.^5^ The conversion of DTNB to TNB by adding GSH was attributed to the shift in the maximum wavelength. The DTNB + GSH + Cu group had the lowest absorbance at 409 nm, owing to GSH depletion by copper ions and further prevention of TNB formation. Moreover, the DTNB + GSH + CNC/LAP group had an absorbance pattern similar to that of the DTNB + GSH group owing to the absence of copper ions. The absorbance value of the DTNB + GSH + CNC/GOx/Cu/LAP group was intermediate to those of the DTNB + GSH and DTNB + GSH + Cu groups. This implies that a certain number of copper ions was involved in GSH depletion; however, the remaining ions were possibly responsible for the interactions with the CNC/LAP structure. Reaction time‐dependent decreasing absorbance pattern (at 409 nm) was seen in DTNB + GSH + CNC/GOx/Cu/LAP group (Figure S10). More copper ions seemed to consume GSH and inhibit the conversion of DTNB to TNB in longer reaction time. The ICG‐incorporated structure group (i.e., CNC/ICG/GOx/Cu/LAP) was excluded from several ROS evaluation assays (Figures 3d,e, S10, and S12) to eliminate interference in absorbance measurements. This finding indicates that the incorporated copper ions can reduce the GSH level in cancer cells and further decrease the hydroxyl radical scavenging effect, thereby leading to improved cellular apoptosis.

Generation of hydroxyl radicals based on the “cascade effect” in the designed system was demonstrated using the TMB assay with glucose (Figure 3e). In addition, the capacity of GOx/Cu in producing sequential hydroxyl radicals from glucose in the gel network was tested in this assay. GOx can produce H_2_O_2_ from glucose, and Cu^+^ can generate hydroxyl radicals from H_2_O_2_ via a Fenton‐like reaction. The hydroxyl radical produced may oxidize TMB (colorless) to its charge‐transfer complex form (blue).^64^ As shown in Figure 3e, the absorbance of the GOx/Cu group increased owing to the cascade generation of hydroxyl radicals and the formation of the blue product of TMB. However, the CNC/LAP group did not display any change in absorbance data owing to the absence of ROS generation potential. Moreover, the CNC/GOx/Cu/LAP group displayed similar absorbance data at 360 min as the GOx/Cu group. This indicates the proper function of ROS production in the gel structures.

Cascade production of hydroxyl radical by GOx/Cu combination was further studied with ESR spectroscopy analysis (Figure S11). With the presence of glucose, the characteristic peak (1:2:2:1) of hydroxyl radical is produced from CNC/ICG/GOx/Cu/LAP gel. Glucose was decomposed to H_2_O_2_ by GOx and it was further converted to hydroxyl radical by copper ion. ESR data also supported the successful catalytic function of designed CNC/ICG/GOx/Cu/LAP gel system.

The H_2_O_2_ generation capability of GOx (introduced into the gel network) was assessed using a TiOSO_4_‐based test (Figure S12). GOx decomposes d‐glucose into H_2_O_2_ and d‐glucuno‐δ‐lactone, and the generated H_2_O_2_ can react with TiOSO_4_ for color development.^65^ The CNC/GOx/LAP group displayed higher absorbance values in the _D_‐glucose (100 mM) group than in the DW group, probably because of the generation of H_2_O_2_ by GOx. Interestingly, there was a significant difference between the CNC/GOx/Cu/LAP and CNC/GOx/LAP groups treated with _D_‐glucose (*p* < 0.05). The copper ions in the CNC/GOx/Cu/LAP group consumed H_2_O_2_, thus the absorbance of the CNC/GOx/Cu/LAP group was lower than that of the CNC/GOx/LAP group. This indicates that the normal enzymatic activity of GOx entrapped in the gel network was maintained.

Cu release from the CNC/ICG/GOx/Cu/LAP structure was investigated (Figure S13). The release rate of Cu at 24 h was 10.2%, which implied a low initial burst release pattern of Cu from the fabricated gel structure. The release rates of Cu on day 14 and 28 were 25.3% and 58.4%, respectively, indicating a sustained release profile of Cu from the CNC/ICG/GOx/Cu/LAP structure. The released copper ions will accumulate in cancer cells and they may contribute to the Fenton‐like reaction‐involved hydroxyl radical generation, which can be used for CDT.

ICG release from the gel system and subsequent entry into cancer cells were assessed by NIRF imaging (Figure S14). To avoid the stability issue of ICG in some types of aqueous buffers,^66^ combined release and cellular accumulation profiles were acquired using NIRF imaging analysis. It exhibited a continuously increasing pattern for 24 h, indicating a high PTT efficiency following intratumoral injection of the developed gel system.

The GOx release profile from the gel system was evaluated by measuring the fluorescence intensity of the FITC‐GOx (Figure S15). Owing to the low sensitivity of the analytical method and the low GOx content in the CNC/ICG/GOx/Cu/LAP structure, a higher amount of FITC‐GOx was included in the gel system for this assay. The release rate of FITC‐GOx from the gel system was 53% at 120 h, indicating a sustained release pattern of GOx. The hybrid CNC/LAP gel structure appeared to affect the controlled release of FITC‐GOx.

### 3.2 In vitro anticancer potentials

**FIGURE 4:**
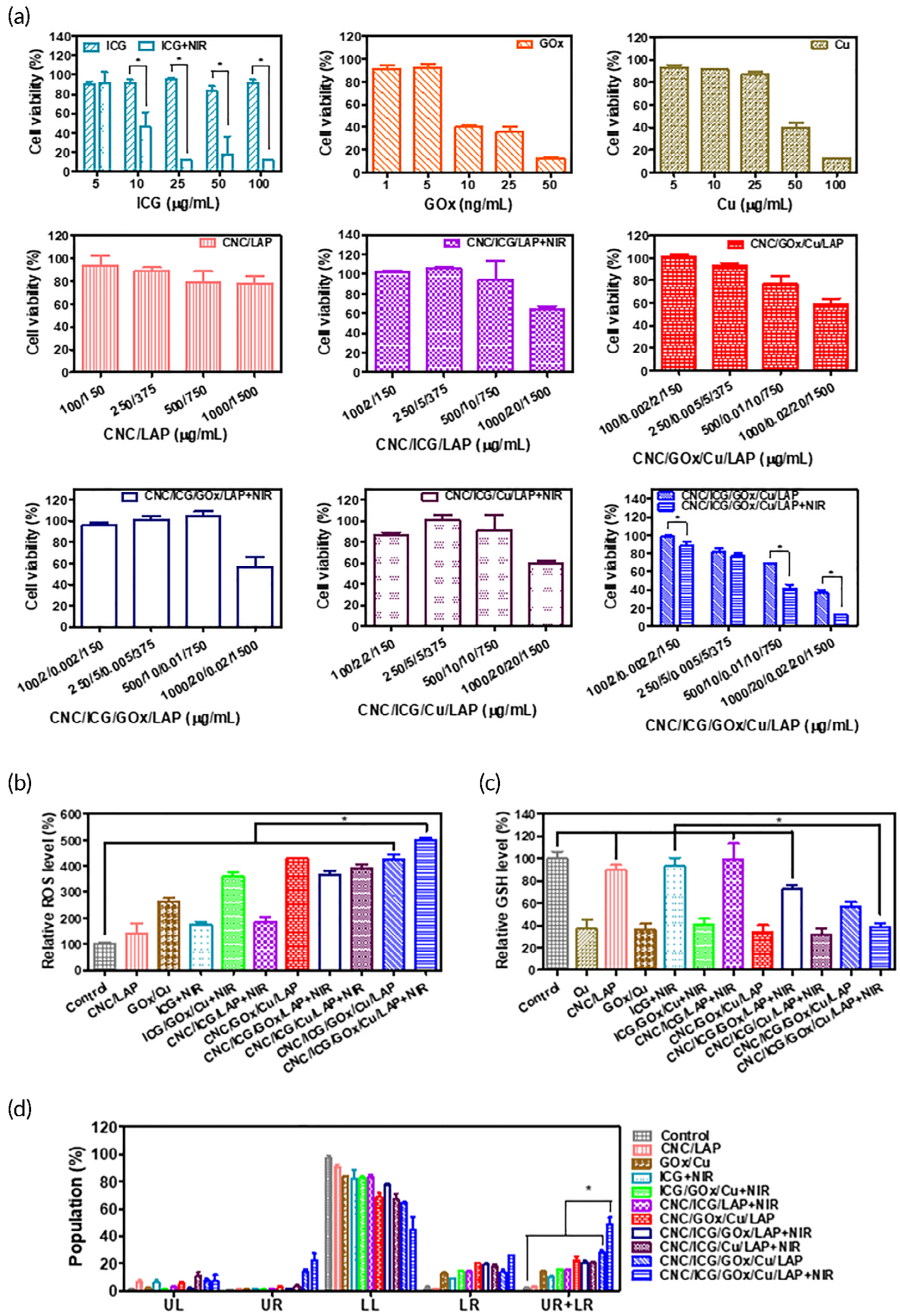
In vitro anticancer efficacies in CT‐26 cells. (a) Antiproliferation assay of ICG (with or without NIR laser), GOx, Cu, CNC/LAP, CNC/ICG/LAP (with NIR laser), CNC/GOx/Cu/LAP, CNC/ICG/GOx/LAP (with NIR laser), CNC/ICG/Cu/LAP (with NIR laser), and CNC/ICG/GOx/Cu/LAP (with or without NIR laser) groups measured by MTS‐based assay. Each point represents mean ± SD (*n* = 4). *p < 0.05, between two groups. (b) Intracellular ROS assay of control, CNC/LAP, GOx/Cu, ICG + NIR, ICG/GOx/Cu + NIR, CNC/ICG/LAP + NIR, CNC/GOx/Cu/LAP, CNC/ICG/GOx/LAP + NIR, CNC/ICG/Cu/LAP + NIR, CNC/ICG/GOx/Cu/LAP, and CNC/ICG/GOx/Cu/LAP + NIR groups. Each point represents mean ± SD (*n* = 3). *p < 0.05, between indicated groups. (c) Intracellular GSH assay of control, Cu, CNC/LAP, GOx/Cu, ICG + NIR, ICG/GOx/Cu + NIR, CNC/ICG/LAP + NIR, CNC/GOx/Cu/LAP, CNC/ICG/GOx/LAP + NIR, CNC/ICG/Cu/LAP + NIR, CNC/ICG/GOx/Cu/LAP, and CNC/ICG/GOx/Cu/LAP + NIR groups. Each point represents mean ± SD (*n* = 4). *p < 0.05, between indicated groups. (d) Apoptosis test of control, CNC/LAP, GOx/Cu, ICG + NIR, ICG/GOx/Cu + NIR, CNC/ICG/LAP + NIR, CNC/GOx/Cu/LAP, CNC/ICG/GOx/LAP + NIR, CNC/ICG/Cu/LAP + NIR, CNC/ICG/GOx/Cu/LAP, and CNC/ICG/GOx/Cu/LAP + NIR groups. Each point represents mean ± SD (*n* = 3). *p < 0.05, between indicated groups.

Antiproliferation efficacy was evaluated by a colorimetric assay in a murine colorectal carcinoma cell line (CT‐26 cell) (Figure 4a). ICG, which has been used as a photothermal agent in the literature,^67^ exhibited concentration‐dependent antiproliferation with NIR light exposure, and significant differences were observed between the NIR‐positive and NIR‐negative groups at 10, 25, 50, and 100 μg/ml (*p* < 0.05). Considering that GOx consumes the accumulated glucose in cancer cells and it leads to cancer cell starvation,^68^ GOx concentration‐dependent antiproliferation profiles were observed in CT‐26 cells, as expected. In addition, Cu was introduced as a Fenton‐like catalyst in this study to provide antiproliferation effects in cancer cells.^5^, ^36^, ^69^, ^70^, ^71^ Higher Cu concentrations resulted in lower cell viability, indicating its antiproliferation potential in CT‐26 cells. Furthermore, glucose is consumed by GOx in cancer cells to produce H_2_O_2_,^37^, ^72^ and the generated H_2_O_2_ can react with copper ions (Fenton‐like reactions), leading to the production of hydroxyl radicals. This cascade reaction may support the role of ST and CDT in cancer treatment. CNC/ICG/LAP + NIR group data suggest concentration‐dependent antiproliferation potential due to PTT efficacy. CNC/GOx/Cu/LAP group has lower cell viability values in higher sample concentration range which may be induced by ST/CDT combination. In CNC/ICG/GOx/LAP + NIR group, marked reduction in cell viability was seen in high sample concentration, indicating the efficacy of PTT/ST. Similar antiproliferation efficacy was observed in CNC/ICG/Cu/LAP + NIR group which may be from PTT and partial CDT options. The CNC/ICG/GOx/Cu/LAP + NIR group exhibited an enhanced antiproliferation effect in the 100/2/0.002/2/150, 500/10/0.01/10/750, and 1000/20/0.02/20/1500 μg/ml groups, compared with the CNC/ICG/GOx/Cu/LAP group (*p* < 0.05). Considering the cell viability data of the CNC/LAP group, the antiproliferation effect seems to be supplied by the ICG/GOx/Cu + NIR combination. These findings indicate that the triple combination of ST/CDT (by GOx/Cu) and PTT (by ICG), rather than partial combination, can provide significantly enhanced antiproliferation effects in CT‐26 cells.

Intracellular ROS levels were measured using the cellular fluorescence intensity (Figure 4b). Measured ROS level of ICG/GOx/Cu + NIR group (aiming at PTT/ST/CDT) was higher than those of GOx/Cu (ST/CDT) and ICG + NIR (PTT) groups, implying the superior ROS generation efficiency of triple combination of therapeutic modalities. Notably, the fluorescence intensity of the CNC/ICG/GOx/Cu/LAP + NIR group was significantly higher than that of CNC/ICG/LAP + NIR (PTT), CNC/GOx/Cu/LAP (ST/CDT), CNC/ICG/GOx/LAP + NIR (PTT/ST), CNC/ICG/Cu/LAP + NIR (PTT/partial CDT), and CNC/ICG/GOx/Cu/LAP (ST/CDT) groups (*p* < 0.05). These findings indicate that the CNC/ICG/GOx/Cu/LAP gel, when combined with NIR light, accelerates glucose deprivation and hydroxyl radical generation. Higher cellular ROS level in the CNC/ICG/GOx/Cu/LAP + NIR group, compared with the other groups, indicates the maximized local anticancer efficacy based on the integrated PTT/ST/CDT strategy in colorectal cancer cells.

The intracellular GSH depletion ability of the designed nanocomposite‐based gel construct was evaluated using colorimetric analysis (Figure 4c).^5^ Intracellular GSH can scavenge hydroxyl radicals; thus, a high GSH depletion capability may be related to its strong anticancer efficacy. The relative GSH level in the CNC/ICG/GOx/Cu/LAP + NIR group was lower than control, CNC/LAP, ICG + NIR, CNC/ICG/LAP + NIR, and CNC/ICG/GOx/LAP + NIR groups (without Cu) (*p* < 0.05). It means that incorporated Cu molecule works appropriately for the conversion of GSH to GSSG. The relative GSH level in the CNC/ICG/GOx/Cu/LAP + NIR group (38.3%) was also lower than that in the CNC/ICG/GOx/Cu/LAP group (57.2%) (*p* < 0.05). Hyperthermia induction can contribute to elevated GSH consumption, as reported.^38^, ^73^, ^74^ It may reduce the ROS scavenging efficacy of GSH and further amplify ROS‐related anticancer activity.

ATP assay was conducted for assessing the catalytic potential of designed hybrid gel system (Figure S16). GOx‐treated group exhibited distinct reduction of intracellular ATP level compared to control (no treatment) group.^75^ CNC/ICG/GOx/Cu/LAP + NIR group also displayed significant decrease in the intracellular ATP level by the glucose deprivation of GOx. It suggests that the bioactivity of incorporated GOx in the gel structure is appropriately maintained for ST. Of note, the combination of CDT/PTT with ST (as shown in CNC/ICG/GOx/Cu/LAP + NIR group) further reduced intracellular ATP level as reported.^76^

The apoptosis‐inducing capability of the developed gel structure system was tested using annexin V and PI‐based assays (Figure 4d). The population in the lower right (LR) and upper right (UR) panels indicates the ratios of early and late apoptosis phases; therefore, the sum value was used for the assessment of apoptosis induction capability in this study. The CNC/LAP group had a similar percentage of (LR + UR) panels compared with the control group, indicating that the CNC/LAP structure has a negligible effect on apoptosis induction under the current experimental conditions. The percentage of (LR + UR) panel in CNC/ICG/GOx/Cu/LAP + NIR group (PTT/ST/CDT) was significantly higher than those of CNC/ICG/LAP + NIR (PTT), CNC/GOx/Cu/LAP (ST/CDT), CNC/ICG/GOx/LAP + NIR (PTT/ST), CNC/ICG/Cu/LAP + NIR (PTT/partial CDT), and CNC/ICG/GOx/Cu/LAP (ST/CDT) groups (*p* < 0.05). The introduction of GOx/Cu to CNC/LAP system significantly elevated the apoptosis potential via a cascade of hydroxyl radical production. Moreover, photothermal conversion by ICG with NIR light absorption (shown in the CNC/ICG/GOx/Cu/LAP + NIR group) also strengthened the apoptosis induction capability, as revealed by the antiproliferation, intracellular ROS, and intracellular GSH assays (Figure 4a−c).

### 3.3 Photothermal characteristics

**FIGURE 5:**
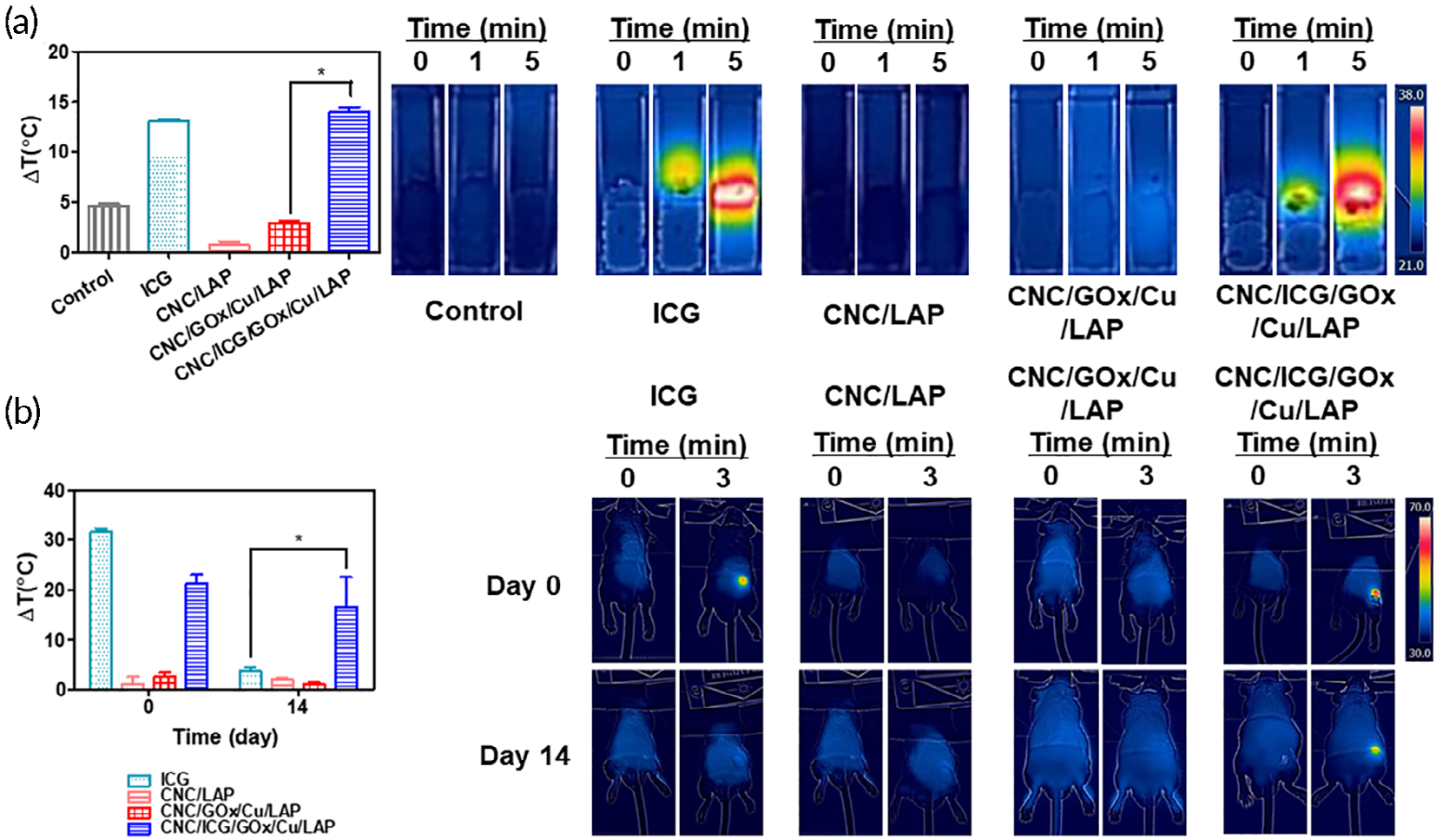
Photothermal activity tests. (a) In vitro photothermal test of control, ICG, CNC/LAP, CNC/GOx/Cu/LAP, and CNC/ICG/GOx/Cu/LAP groups. Temperature change degrees are plotted and thermal camera images are displayed. Each point represents mean ± SD (*n* = 3). *p < 0.05, between two groups. (b) In vivo photothermal test of ICG, CNC/LAP, CNC/GOx/Cu/LAP, and CNC/ICG/GOx/Cu/LAP groups following subcutaneous injection in mouse. Temperature change degrees (on day 0 and 14) are plotted and thermal camera images (on day 0 and 14) are displayed. Each point represents mean ± SD (*n* = 4). *p < 0.05, between two groups.

The photothermal efficiency of the developed formulation was assessed through in vitro and in vivo experiments (Figure 5). As shown in Figure 5a, ICG was recognized as a photothermal organic agent, and the ICG group displayed a 13.1 °C increase under NIR laser irradiation for 5 min. The CNC/ICG/GOx/Cu/LAP group was subjected to a temperature elevation of 14.0 °C following 5 min of NIR laser absorption. As the CNC/LAP and CNC/GOx/Cu/LAP groups did not achieve sufficient temperature elevation, ICG seemed to act as a key modality of PTT in this study. The time‐dependent photothermal transducing activity was also confirmed in the animal test (Figure 5b). The ICG and CNC/ICG/GOx/Cu/LAP groups showed a temperature elevation of >20 °C on day 0, which is sufficient for inducing hyperthermia in localized cancer tissue. Interestingly, even on day 14, CNC/ICG/GOx/Cu/LAP group still had a 16.6 °C of temperature increment while the ICG group exhibited only a 3.6 °C increase, probably due to the fast diffusion rate of ICG solution into the adjacent tissues. The encapsulated ICG in the gel remained on day 14 owing to the slow degradation rate of the gel, thereby eliminating the necessity of multiple injections of the gel formulation. Furthermore, ICG loading into the CNC/LAP structure may provide a long‐acting delivery profile following local extravascular injection. The persistent photothermal transducing characteristics of the CNC/ICG/GOx/Cu/LAP gel would be very effective for cancer therapy without multiple dosing.

### 3.4 Biodegradation features

**FIGURE 6:**
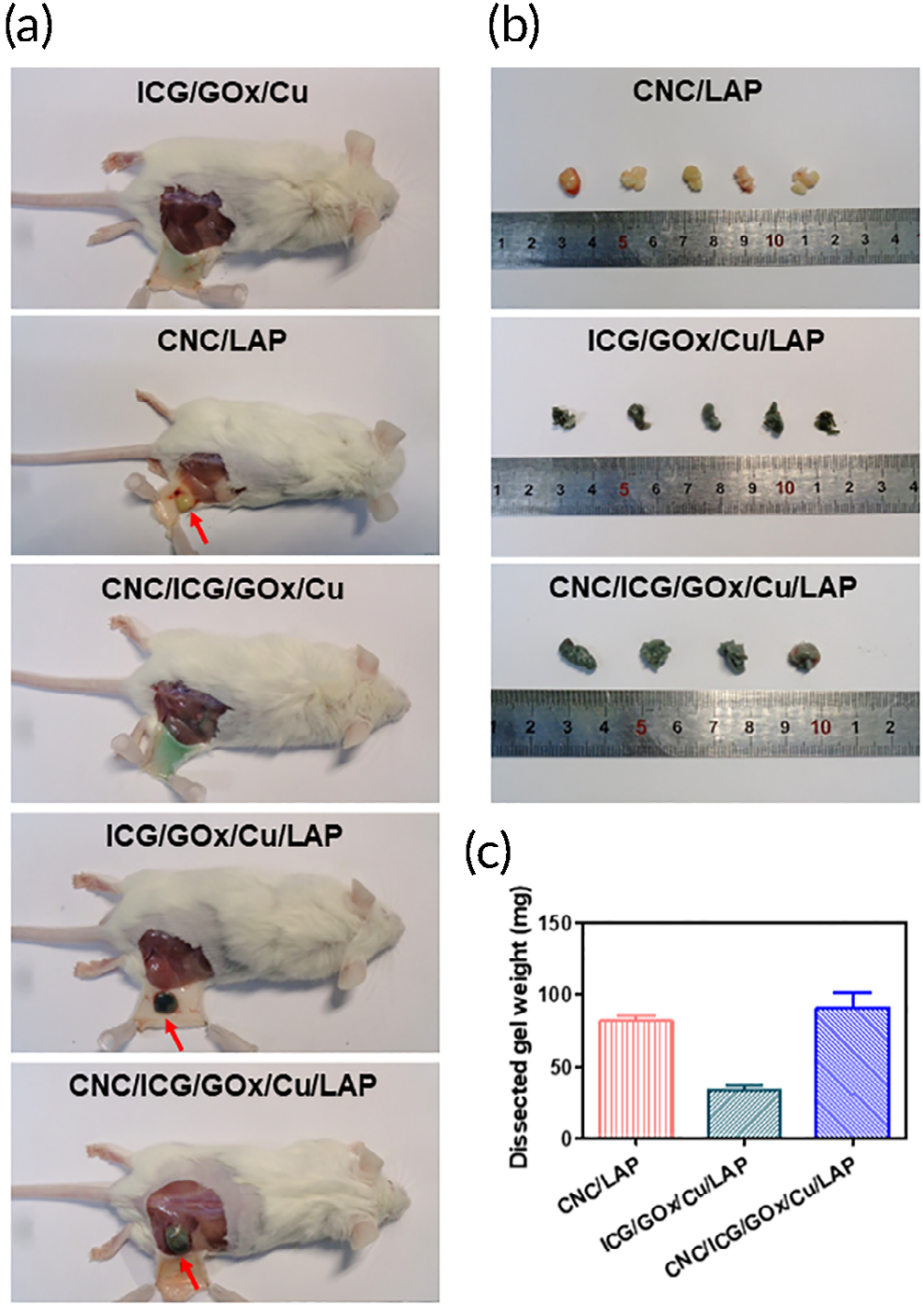
Biodegradation test of designed gel systems in mouse. (a) Images of gel‐injected site in ICG/GOx/Cu, CNC/LAP, CNC/ICG/GOx/Cu, ICG/GOx/Cu/LAP, and CNC/ICG/GOx/Cu/LAP groups. Red arrow indicates the remained specimen. (b) Images of excised gel mass from CNC/LAP, ICG/GOx/Cu/LAP, and CNC/ICG/GOx/Cu/LAP groups. (c) Dissected gel weight of CNC/LAP, ICG/GOx/Cu/LAP, and CNC/ICG/GOx/Cu/LAP groups. Each point represents mean ± SD (*n* ≥ 4).

The biodegradation features of the prepared gel structures were estimated in mice following subcutaneous injection (Figure 6). Residual sample fragments were not obtained from the ICG/GOx/Cu and CNC/ICG/GOx/Cu groups, which may have resulted from low viscosity‐related immediate diffusion and dissociation in the subcutaneous tissue. The remaining amounts of the CNC/LAP and CNC/ICG/GOx/Cu/LAP groups were higher than those of the ICG/GOx/Cu/LAP group which may be due to the existence of CNC and the interactions between CNC and LAP. Residual ratios, which can be presented as dissected mass weight to initial injected sample weight, of CNC/LAP and CNC/ICG/GOx/Cu/LAP groups were also higher than that of ICG/GOx/Cu/LAP group. Interactions between CNC and LAP dispersed in the gel network may contribute to the slow biodegradation rate.

### 3.5 Systemic toxicity

**FIGURE 7:**
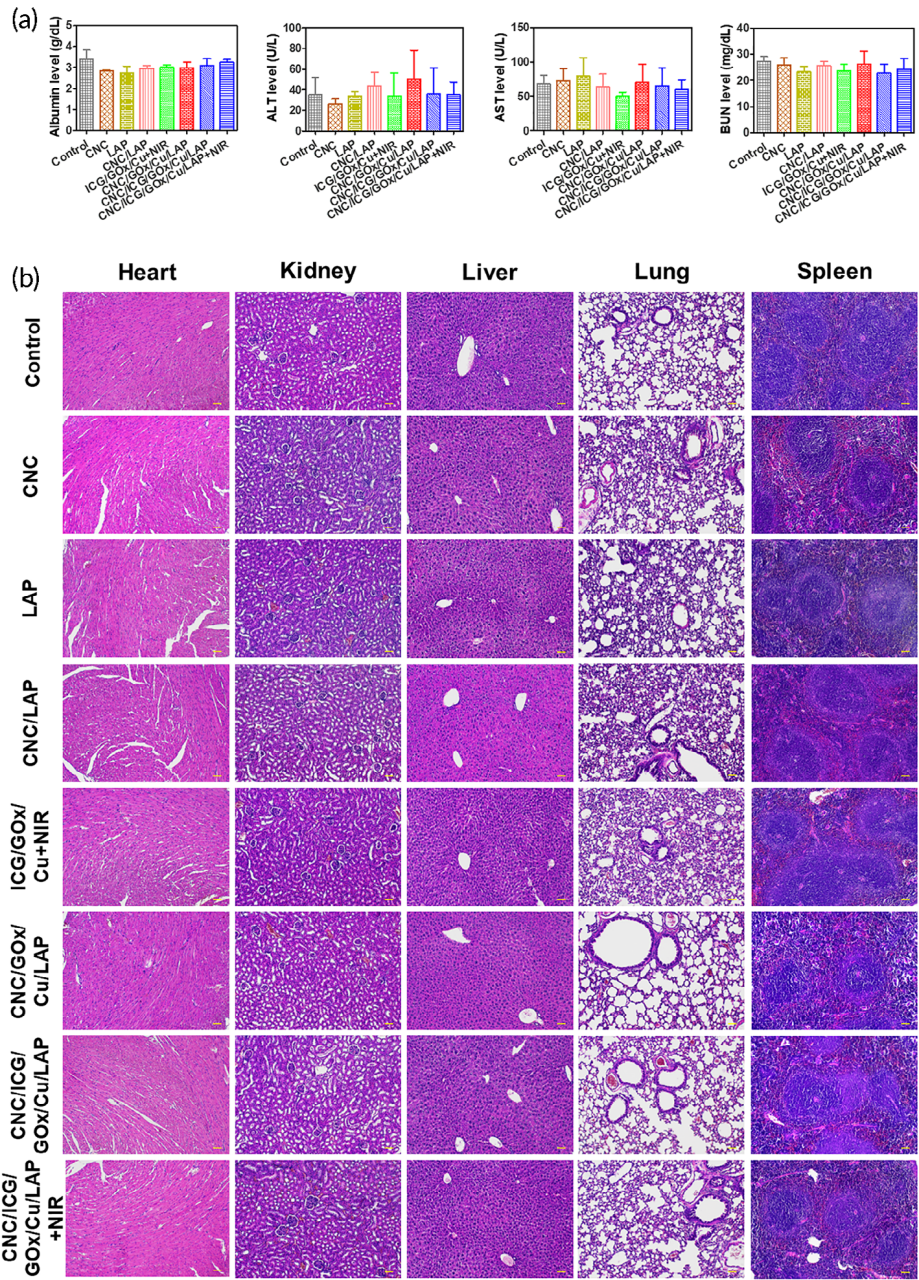
Systemic toxicity tests of developed gel systems. (a) Blood chemistry data of control, CNC, LAP, CNC/LAP, ICG/GOx/Cu + NIR, CNC/GOx/Cu/LAP, CNC/ICG/GOx/Cu/LAP, and CNC/ICG/GOx/Cu/LAP + NIR groups. Albumin, ALT, AST, and BUN levels in serum are plotted. Each point represents mean ± SD (*n* = 5). (b) H&E images of control, CNC, LAP, CNC/LAP, ICG/GOx/Cu + NIR, CNC/GOx/Cu/LAP, CNC/ICG/GOx/Cu/LAP, and CNC/ICG/GOx/Cu/LAP + NIR groups. Histological images of heart, kidney, liver, lung, and spleen are shown. Scale bar = 100 μm.

The systemic toxicity of the nanoconstruct‐aligned gel structure was evaluated using blood biochemistry and histological tests (Figure 7). CNC, LAP, and CNC/LAP groups did not obviously increase the serum levels of markers compared to control group. As shown in Figure 7a, the CNC/ICG/GOx/Cu/LAP group did not show significant differences in albumin, ALT, AST, and BUN levels compared with the control group. NIR laser exposure in the CNC/ICG/GOx/Cu/LAP group also did not induce any differences in all serum markers compared to the control group. This suggests that CNC/ICG/GOx/Cu/LAP combined with NIR laser irradiation did not induce fatal hepatic or renal toxicity. In H&E‐stained images of the heart, kidney, liver, lung, and spleen (Figure 7b), CNC, LAP, and CNC/LAP groups did not show any significant pathological changes compared to control group, implying their safe application via subcutaneous route. There was no definite pathological difference between the control and CNC/ICG/GOx/Cu/LAP groups and NIR laser application to the CNC/ICG/GOx/Cu/LAP‐injected site also did not produce any histopathological changes compared with the control group. These findings indicate the safe application of the designed CNC/ICG/GOx/Cu/LAP gel structure in combination with NIR light.

### 3.6 Local anticancer properties

**FIGURE 8:**
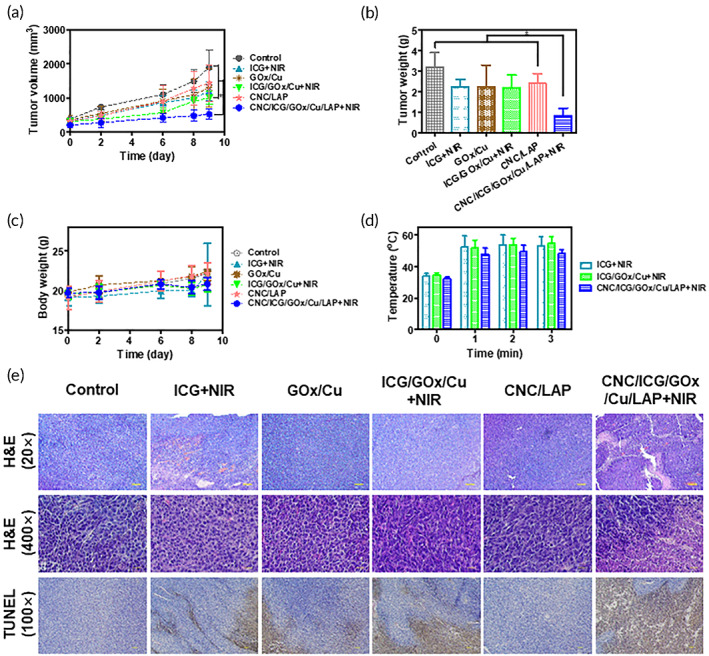
In vivo anticancer activity tests of developed gel systems. (a) Tumor growth profiles of control, ICG + NIR, GOx/Cu, ICG/GOx/Cu + NIR, CNC/LAP, and CNC/ICG/GOx/Cu/LAP + NIR groups. Each point represents mean ± SD (*n* = 5–7). *p < 0.05, between indicated groups. (b) Dissected tumor weight data of control, ICG + NIR, GOx/Cu, ICG/GOx/Cu + NIR, CNC/LAP, and CNC/ICG/GOx/Cu/LAP + NIR groups. Each point represents mean ± SD (*n* = 5–7). *p < 0.05, between indicated groups. (c) Body weight profiles of control, ICG + NIR, GOx/Cu, ICG/GOx/Cu + NIR, CNC/LAP, and CNC/ICG/GOx/Cu/LAP + NIR groups. Each point represents mean ± SD (*n* = 5–7). (d) Temperature change profiles of ICG + NIR, ICG/GOx/Cu + NIR, and CNC/ICG/GOx/Cu/LAP + NIR groups. Each point represents mean ± SD (*n* = 6). (e) H&E and TUNEL images of control, ICG + NIR, GOx/Cu, ICG/GOx/Cu + NIR, CNC/LAP, and CNC/ICG/GOx/Cu/LAP + NIR groups. Scale bar length: 20 μm (400× magnification), 50 μm (100× magnification), and 100 μm (20× magnification).

The therapeutic potential of CNC/ICG/GOx/Cu/LAP combined with NIR laser was assessed in a mouse model following intratumoral injection (Figures 8 and S17). The ICG + NIR, GOx/Cu, and ICG/GOx/Cu + NIR groups were selected to evaluate the PTT, ST/CDT, and PTT/ST/CDT potentials in this study. The combined therapeutic potential of the CNC/ICG/GOx/Cu/LAP + NIR group was assessed and compared to that of the blank gel (CNC/LAP) group. On the final day, the tumor volume in the CNC/ICG/GOx/Cu/LAP + NIR group was 27.7% of that in the control group, which was lower than those in the other groups (*p* < 0.05; Figure 8a). Accordingly, the excised tumor weight of the CNC/ICG/GOx/Cu/LAP + NIR group was lower than that of the other groups, at 26.6% of that of the control group (*p* < 0.05; Figure 8b). It indicates the superior tumor growth suppressive efficiency of ICG/GOx/Cu‐installed CNC/LAP gel structure by combination of PTT, ST, and CDT approaches. Sustained release of drug cargos and longer in vivo retention of gel system may explain the superior anticancer activity of CNC/ICG/GOx/Cu/LAP + NIR group. The tumor growth inhibition potential of the CNC/ICG/GOx/Cu/LAP + NIR group was clearly demonstrated by tumor volume and weight measurements (Figure 8a,b). There was also no significant difference in body weight between the CNC/ICG/GOx/Cu/LAP + NIR group and the other groups during the monitoring period, suggesting the absence of severe systemic toxicity of the fabricated gel system with NIR absorption (Figure 8c). Upon NIR light absorption, the temperature at the injection site was elevated in the ICG, ICG/GOx/Cu, and CNC/ICG/GOx/Cu/LAP groups (Figures 8d and S18). Notably, the CNC/ICG/GOx/Cu/LAP group achieved a temperature increase to 48 °C, which is appropriate for the induction of hyperthermia in cancer.^35^ The apoptosis induction potential of the designed gel system was evaluated by histological assays (Figure 8e). The apoptosis induction potential of the CNC/ICG/GOx/Cu/LAP + NIR group was higher than those of the other groups, which is in good agreement with the results of the annexin V and PI‐based assays in the cell culture model (Figure 4d). Local injection of the CNC/ICG/GOx/Cu/LAP gel system combined with NIR laser irradiation resulted in a high tumor‐growth inhibitory effect with minimal toxicity. Colorectal cancer is one of easily accessed cancer types via an endoscopic approach. Thus, we believe that developed gel system can be applied for reducing tumor size prior to surgery and inhibiting its recurrence after surgery.

## 4 CONCLUSIONS

In this study, organic nanorod (CNC) and inorganic nanodisk (LAP) hybridized gel systems were designed to realize glucose deprivation in cancer cells (for ST), a self‐supplying cascade of hydroxyl radicals (for CDT), and light‐to‐heat conversion (for PTT). Nano‐size particle‐aligned gel structures were designed by optimizing the concentrations of CNC and LAP to tune the rheological properties. As modalities of ST and CDT, GOx and Cu were introduced to the CNC/LAP structure for glucose decomposition and Fenton‐like reaction‐based hydroxyl radical production, respectively. Furthermore, Cu contributed to cellular GSH depletion and enhanced anticancer activity. ICG was entrapped in the CNC/LAP structure to induce hyperthermia in the tumor tissue upon NIR laser irradiation. However, it not only achieved its objective but also further amplified the CDT efficacy of the GOx/Cu combination. In short, the CNC/LAP gel system exhibited higher retention capability at the injection site, and a prolonged PTT/ST/CDT effect was achieved. Thus, the results of this study indicate that the designed nano‐unit‐aligned gel network is effective for local cancer therapy and can ensure a high safety profile.

## AUTHOR CONTRIBUTIONS

**Sungyun Kim:** Conceptualization (equal); formal analysis (lead); investigation (lead); visualization (equal); writing – original draft (lead); writing – review and editing (equal). **ChaeRim Hwang:** investigation, formal analysis, visualization, writing—original draft. **Da In Jeong:** investigation, formal analysis. **JiHye Park:** investigation, formal analysis. **Han‐Jun Kim:** investigation, formal analysis. **KangJu Lee:** investigation, formal analysis. **Junmin Lee:** investigation, formal analysis. **Seung‐Hwan Lee:** Funding acquisition (lead); resources (equal); writing – review and editing (supporting). **Hyun‐Jong Cho:** conceptualization, supervision, funding acquisition, resources, writing—original draft, writing—review & editing.

## CONFLICT OF INTEREST

The authors declare no conflict of interest.

### PEER REVIEW

The peer review history for this article is available at https://publons.com/publon/10.1002/btm2.10470.

## Supporting information


**APPENDIX S1.** Supporting information

## Data Availability

The raw/processed data required to reproduce these findings cannot be shared at this time due to technical or time limitations.

## References

[1] Cho HJ . Recent progresses in the development of hyaluronic acid‐based nanosystems for tumor‐targeted drug delivery and cancer imaging. J Pharm Investig. 2020;50:115‐129.

[2] Choi JW , Lee SY , Cho EJ , et al. Gas generating microspheres for immediate release of Hsp90 inhibitor aiming at postembolization hypoxia in transarterial chemoembolization therapy of hepatocellular carcinoma. Int J Pharm. 2021;607:120988. 34389420 10.1016/j.ijpharm.2021.120988

[3] Gupta B , Kim JO . Recent progress in cancer immunotherapy approaches based on nanoparticle delivery devices. J Pharm Investig. 2021;51:399‐412.

[4] Jeong DI , Kim S , Lee SY , et al. Iron sulfate‐reinforced hydrogel reactors with glucose deprivation, serial reactive oxygen species generation, ferroptosis induction, and photothermal ablation for cancer therapy. Chem Eng J. 2022;438:135584.

[5] Kim S , Seo JH , Jeong DI , et al. Fenton‐like reaction, glutathione reduction, and photothermal ablation‐built‐in hydrogels crosslinked by cupric sulfate for loco‐regional cancer therapy. Biomater Sci. 2021;9:847‐860. 33232388 10.1039/d0bm01470b

[6] Lee SY , Park JH , Ko SH , et al. Mussel‐inspired hyaluronic acid derivative nanostructures for improved tumor targeting and penetration. ACS Appl Mater Interfaces. 2017;9:22308‐22320. 28621523 10.1021/acsami.7b06582

[7] Lee SY , Ko SH , Shim JS , et al. Tumor targeting and lipid rafts disrupting hyaluronic acid‐cyclodextrin‐based nanoassembled structure for cancer therapy. ACS Appl Mater Interfaces. 2018;10:36628‐36640. 30298719 10.1021/acsami.8b08243

[8] Lee SY , Hong EH , Jeong JY , et al. Esterase‐sensitive cleavable histone deacetylase inhibitor‐coupled hyaluronic acid nanoparticles for boosting anticancer activities against lung adenocarcinoma. Biomater Sci. 2019;7:4624‐4635. 31451819 10.1039/c9bm00895k

[9] Melamed JR , Edelstein RS , Day ES . Elucidating the fundamental mechanisms of cell death triggered by photothermal therapy. ACS Nano. 2015;9:6‐11. 25590560 10.1021/acsnano.5b00021

[10] Riley RS , June CH , Langer R , et al. Delivery technologies for cancer immunotherapy. Nat Rev Drug Discov. 2019;18:175‐196. 30622344 10.1038/s41573-018-0006-z PMC6410566

[11] Sharman WM , van Lier JE , Allen CM . Targeted photodynamic therapy via receptor mediated delivery systems. Adv Drug Deliv Rev. 2004;56:53‐76. 14706445 10.1016/j.addr.2003.08.015

[12] Shim G , Jeong S , Oh JL , et al. Lipid‐based nanoparticles for photosensitive drug delivery systems. J Pharm Investig. 2022;52:151‐160. 10.1007/s40005-021-00553-9 PMC8731178 35013696

[13] Tang Z , Liu Y , He M , et al. Chemodynamic therapy: tumour microenvironment‐mediated Fenton and Fenton‐like reactions. Angew Chem Int ed. 2019;58:946‐956. 10.1002/anie.201805664 30048028

[14] Tian Y , Liu Z , Tan H , et al. New aspects of ultrasound‐mediated targeted delivery and therapy for cancer. Int J Nanomedicine. 2020;15:401‐408. 32021187 10.2147/IJN.S201208 PMC6982438

[15] Xie Y , Wang M , Sun Q , et al. PtBi‐β‐CD‐Ce6 nanozyme for combined trimodal imaging‐guided photodynamic therapy and NIR‐II responsive photothermal therapy. Inorg Chem. 2022;61:6852‐6860. 35477242 10.1021/acs.inorgchem.2c00168

[16] Yao J , Zheng F , Yao C , et al. Rational design of nanomedicine for photothermal‐chemodynamic bimodal cancer therapy. Wiley Interdiscip Rev Nanomed Nanobiotechnol. 2021;13:e1682. 33185008 10.1002/wnan.1682

[17] Wu H , Gu D , Xia S , et al. One‐for‐all intelligent core‐shell nanoparticles for tumor‐specific photothermal‐chemodynamic synergistic therapy. Biomater Sci. 2021;9:1020‐1033. 33325928 10.1039/d0bm01734e

[18] Xiao S , Lu Y , Feng M , et al. Multifunctional FeS_2_ theranostic nanoparticles for photothermal‐enhanced chemodynamic/photodynamic cancer therapy and photoacoustic imaging. Chem Eng J. 2020;396:125294.

[19] Choi JW , Park JH , Baek SY , et al. Doxorubicin‐loaded poly(lactic‐co‐glycolic acid) microspheres prepared using the solid‐in‐oil‐in‐water method for the transarterial chemoembolization of a liver tumor. Colloids Surf B Biointerfaces. 2015;132:305‐312. 26057730 10.1016/j.colsurfb.2015.05.037

[20] Deng H , Dong A , Song J , et al. Injectable thermosensitive hydrogel systems based on functional PEG/PCL block polymer for local drug delivery. J Control Release. 2019;297:60‐70. 30684513 10.1016/j.jconrel.2019.01.026

[21] Hwang C , Lee SY , Kim HJ , et al. Polypseudorotaxane and polydopamine linkage‐based hyaluronic acid hydrogel network with a single syringe injection for sustained drug delivery. Carbohydr Polym. 2021;266:118104. 34044922 10.1016/j.carbpol.2021.118104

[22] Kim MH , Park JH , Nguyen DT , et al. Hyaluronidase inhibitor‐incorporated cross‐linked hyaluronic acid hydrogels for subcutaneous injection. Pharmaceutics. 2021;13:170. 33513991 10.3390/pharmaceutics13020170 PMC7910999

[23] Lee SY , Yang M , Seo JH , et al. Serially pH‐modulated hydrogels based on boronate ester and polydopamine linkages for local cancer therapy. ACS Appl Mater Interfaces. 2021;13:2189‐2203. 33416318 10.1021/acsami.0c16199

[24] Peers S , Montembault A , Ladavière C . Chitosan hydrogels for sustained drug delivery. J Control Release. 2020;326:150‐163. 32562854 10.1016/j.jconrel.2020.06.012

[25] Du H , Liu W , Zhang M , et al. Cellulose nanocrystals and cellulose nanofibrils based hydrogels for biomedical applications. Carbohydr Polym. 2019;209:130‐144. 30732792 10.1016/j.carbpol.2019.01.020

[26] Seo JH , Lee SY , Hwang C , et al. Multi‐layered cellulose nanocrystal system for CD44 receptor‐positive tumor‐targeted anticancer drug delivery. Int J Biol Macromol. 2020;162:798‐809. 32585268 10.1016/j.ijbiomac.2020.06.193

[27] Tang J , Sisler J , Grishkewich N , et al. Functionalization of cellulose nanocrystals for advanced applications. J Colloid Interface Sci. 2017;494:397‐409. 28187295 10.1016/j.jcis.2017.01.077

[28] Habibi Y , Lucia LA , Rojas OJ . Cellulose nanocrystals: chemistry, selfassembly, and applications. Chem Rev. 2010;110:3479‐3500. 20201500 10.1021/cr900339w

[29] Tomás H , Alves CS , Rodrigues J . Laponite: a key nanoplatform for biomedical applications? Nanomedicine. 2018;14:2407‐2420. 28552649 10.1016/j.nano.2017.04.016

[30] Tom C , Ganesh Tanuku VMS , Paineau E , et al. Binary mixtures of colloidal cellulose nanocrystals and laponite for preparation of functional nanocomposites. ACS Appl Nano Mater. 2021;4:8586‐8599.

[31] Šebenik U , Lapasin R , Krajnc M . Rheology of aqueous dispersions of laponite and TEMPO‐oxidized nanofibrillated cellulose. Carbohydr Polym. 2020;240:116330. 32475587 10.1016/j.carbpol.2020.116330

[32] Davila JL , d'Ávila MA . Laponite as a rheology modifier of alginate solutions: physical gelation and aging evolution. Carbohydr Polym. 2017;157:1‐8. 27987800 10.1016/j.carbpol.2016.09.057

[33] Zheng X , Xing D , Zhou F , et al. Indocyanine green‐containing nanostructure as near infrared dual‐functional targeting probes for optical imaging and photothermal therapy. Mol Pharm. 2011;8:447‐456. 21197955 10.1021/mp100301t

[34] Fu LH , Qi C , Lin J , et al. Catalytic chemistry of glucose oxidase in cancer diagnosis and treatment. Chem Soc Rev. 2018;47:6454‐6472. 30024579 10.1039/c7cs00891k

[35] Cao C , Wang X , Yang N , et al. Recent advances of cancer chemodynamic therapy based on Fenton/Fenton‐like chemistry. Chem Sci. 2022;13:863‐889. 35211255 10.1039/d1sc05482a PMC8790788

[36] Fu LH , Wan Y , Qi C , et al. Nanocatalytic theranostics with glutathione depletion and enhanced reactive oxygen species generation for efficient cancer therapy. Adv Mater. 2021;33:2006892. 10.1002/adma.202006892 33394515

[37] Wang M , Wang D , Chen Q , et al. Recent advances in glucose‐oxidase‐based nanocomposites for tumor therapy. Small. 2019;15:1903895. 10.1002/smll.201903895 31747128

[38] Sun Q , Liu B , Wang Z , et al. H_2_O_2_/O_2_ self‐supplementing and GSH‐depleting Ca^2+^ nanogenerator with hyperthermia‐triggered, TME‐responsive capacities for combination cancer therapy. Chem Eng J. 2021;425:131485.

[39] Wang M , Chang M , Li C , et al. Tumor‐microenvironment‐activated reactive oxygen species amplifier for enzymatic cascade cancer starvation/chemodynamic/immunotherapy. Adv Mater. 2022;34:2106010. 10.1002/adma.202106010 34699627

[40] Zhang Y , Jiang S , Lin J , et al. Antineoplastic enzyme as drug carrier with activatable catalytic activity for efficient combined therapy. Angew Chem Int Ed. 2022;61:202208583. 10.1002/anie.202208583 35848681

[41] Wang M , Chang M , Chen Q , et al. Au2Pt‐PEG‐Ce6 nanoformulation with dual nanozyme activities for synergistic chemodynamic therapy/phototherapy. Biomaterials. 2020;252:120093. 32422490 10.1016/j.biomaterials.2020.120093

[42] Chang M , Wang M , Wang M , et al. A multifunctional cascade bioreactor based on hollow‐structured Cu_2_MoS_4_ for synergetic cancer chemo‐dynamic therapy/starvation therapy/phototherapy/immunotherapy with remarkably enhanced efficacy. Adv Mater. 2019;31:1905271. 10.1002/adma.201905271 31680346

[43] Thompson DW , Butterworth JT . The nature of laponite and its aqueous dispersions. J Colloid Interface Sci. 1992;151:236‐243.

[44] Zinge C , Kandasubramanian B . Nanocellulose based biodegradable polymers. Eur Polym J. 2020;133:109758.

[45] You C , Ning L , Zhang Z , et al. Toxic reactive oxygen species enhanced chemodynamic therapy by copper metal‐nanocellulose based nanocatalysts. Carbohydr Polym. 2022;289:119432. 35483845 10.1016/j.carbpol.2022.119432

[46] You C , Ning L , Wu H , et al. A biocompatible and pH‐responsive nanohydrogel based on cellulose nanocrystal for enhanced toxic reactive oxygen species generation. Carbohydr Polym. 2021;258:117685. 33593558 10.1016/j.carbpol.2021.117685

[47] Xu F , Liu M , Li X , et al. Loading of indocyanine green within polydopamine‐coated laponite nanodisks for targeted cancer photothermal and photodynamic therapy. Nanomaterials. 2018;8:347. 29783745 10.3390/nano8050347 PMC5977361

[48] Bertsch P , Sánchez‐Ferrer A , Bagnani M , et al. Ion‐induced formation of nanocrystalline cellulose colloidal glasses containing nematic domains. Langmuir. 2019;35:4117‐4124. 30810320 10.1021/acs.langmuir.9b00281

[49] Lee SY , Park JH , Yang M , et al. Ferrous sulfate‐directed dual‐cross‐linked hyaluronic acid hydrogels with long‐term delivery of donepezil. Int J Pharm. 2020;582:119309. 32278055 10.1016/j.ijpharm.2020.119309

[50] Yang M , Lee SY , Kim S , et al. Selenium and dopamine‐crosslinked hyaluronic acid hydrogel for chemophotothermal cancer therapy. J Control Release. 2020;324:750‐764. 32304718 10.1016/j.jconrel.2020.04.024

[51] Kontoudakis N , Smith M , Smith PA , et al. The colorimetric determination of copper in wine: total copper. Aust J Grape Wine Res. 2020;26:121‐129.

[52] Faustino‐Rocha A , Oliveira PA , Pinho‐Oliveira J , et al. Estimation of rat mammary tumor volume using caliper and ultrasonography measurements. Lab Anim. 2013;42:217‐224. 10.1038/laban.254 23689461

[53] Deng Z , Fang C , Ma X , et al. One stone two birds: Zr‐Fc metal‐organic framework nanosheet for synergistic photothermal and chemodynamic cancer therapy. ACS Appl Mater Interfaces. 2020;12:20321‐20330. 32293862 10.1021/acsami.0c06648

[54] Zhong L , Fu S , Peng X , et al. Colloidal stability of negatively charged cellulose nanocrystalline in aqueous systems. Carbohydr Polym. 2012;90:644‐649. 24751088 10.1016/j.carbpol.2012.05.091

[55] Yuan Z , Fan Q , Dai X , et al. Cross‐linkage effect of cellulose/laponite hybrids in aqueousdispersions and solid films. Carbohydr Polym. 2014;102:431‐437. 24507302 10.1016/j.carbpol.2013.11.051

[56] Silva JM , Barud HS , Meneguin AB , et al. Inorganic‐organic bio‐nanocomposite films based on laponite and cellulose nanofibers (CNF). Appl Clay Sci. 2019;168:428‐435.

[57] Brito BSL , Pereira FV , Putaux JL , et al. Preparation, morphology and structure of cellulose nanocrystals from bamboo fibers. Cellul. 2012;19:1527‐1536.

[58] Wu Z , Xu J , Gong J , et al. Preparation, characterization and acetylation of cellulose nanocrystal allomorphs. Cellulose. 2018;25:4905‐4918.

[59] Cunha VRR , Lima FCDA , Sakai VY , et al. LAPONITE‐pilocarpine hybrid material: experimental and theoretical evaluation of pilocarpine conformation. RSC Adv. 2017;7:27290‐27298.

[60] Munier P , Di A , Hadi SE , et al. Assembly of cellulose nanocrystals and clay nanoplatelets studied by time‐resolved X‐ray scattering. Soft Matt. 2021;17:5747‐5755. 10.1039/d1sm00251a 34019065

[61] Bellani CF , Pollet E , Hebraud A , et al. Morphological, thermal, and mechanical properties of poly(ε‐caprolactone)/poly(ε‐caprolactone)‐grafted‐cellulose nanocrystals mats produced by electrospinning. J Appl Polym Sci. 2016;133:43445.

[62] Bertsch P , Isabettini S , Fischer P . Ion‐induced hydrogel formation and nematic ordering of nanocrystalline cellulose suspensions. Biomacromolecules. 2017;18:4060‐4066. 29028331 10.1021/acs.biomac.7b01119

[63] Rahman I , Kode A , Biswas SK . Assay for quantitative determination of glutathione and glutathione disulfide levels using enzymatic recycling method. Nat Protoc. 2006;1:3159‐3165. 17406579 10.1038/nprot.2006.378

[64] Song H , Li X , He Y , et al. Colorimetric evaluation of the hydroxyl radical scavenging ability of antioxidants using carbon‐confined CoO_x_ as a highly active peroxidase mimic. Mikrochim Acta. 2019;186:354. 31098776 10.1007/s00604-019-3488-4

[65] Deadman BJ , Hellgardt K , Hii KK . A colorimetric method for rapid and selective quantification of peroxodisulfate, peroxomonosulfate and hydrogen peroxide. React Chem Eng. 2017;2:462‐466.

[66] Saxena V , Sadoqi M , Shao J . Degradation kinetics of indocyanine green in aqueous solution. J Pharm Sci. 2003;92:2090‐2097. 14502548 10.1002/jps.10470

[67] Porcu EP , Salis A , Gavini E , et al. Indocyanine green delivery systems for tumour detection and treatments. Biotechnol Adv. 2016;34:768‐789. 27090752 10.1016/j.biotechadv.2016.04.001

[68] Fu LH , Qi C , Hu YR , et al. Glucose oxidase‐instructed multimodal synergistic cancer therapy. Adv Mater. 2019;31:1808325. 10.1002/adma.201808325 30907460

[69] Chudal L , Pandey NK , Phan J , et al. Copper‐cysteamine nanoparticles as a heterogeneous Fenton‐like catalyst for highly selective cancer treatment. ACS Appl Bio Matter. 2020;3:1804‐1814. 10.1021/acsabm.0c00098 35021670

[70] Dong C , Feng W , Xu W , et al. The coppery age: copper (Cu)‐involved nanotheranostics. Adv Sci. 2020;7:2001549. 10.1002/advs.202001549 PMC7610332 33173728

[71] Koo JS , Lee SY , Nam S , et al. Preparation of cupric sulfate‐based self‐emulsifiable nanocomposites and their application to the photothermal therapy of colon adenocarcinoma. Biochem Biophys Res Commun. 2018;503:2471‐2477. 30208513 10.1016/j.bbrc.2018.07.002

[72] Guo Y , Jia HR , Zhang X , et al. A glucose/oxygen‐exhausting nanoreactor for starvation‐ and hypoxia‐activated sustainable and cascade chemo‐chemodynamic therapy. Small. 2020;16:2000897. 10.1002/smll.202000897 32537936

[73] Dong S , Dong Y , Jia T , et al. GSH‐depleted nanozymes with hyperthermia‐enhanced dual enzyme‐mimic activities for tumor nanocatalytic therapy. Adv Mater. 2020;32:2002439. 10.1002/adma.202002439 32914495

[74] Hao Y , Dong Z , Chen M , et al. Near‐infrared light and glucose dual‐responsive cascading hydroxyl radical generation for in situ gelation and effective breast cancer treatment. Biomaterials. 2020;228:119568. 31677393 10.1016/j.biomaterials.2019.119568

[75] Wang X , Li Y , Jia F , et al. Boosting nutrient starvation‐dominated cancer therapy through curcumin‐augmented mitochondrial Ca^2+^ overload and obatoclax‐mediated autophagy inhibition as supported by a novel nano‐modulator GO‐Alg@CaP/CO. J Nanobiotechnol. 2022;20:225. 10.1186/s12951-022-01439-0 PMC9097046 35551609

[76] Xu R , Zhang D , Tan J , et al. A multifunctional cascade bioreactor based on a layered double oxides composite hydrogel for synergetic tumor chemodynamic/starvation/photothermal therapy. Acta Biomater. 2022;153:494‐504. 36115653 10.1016/j.actbio.2022.09.024

