# Supplementary material for: Nanorod/nanodisk‐integrated liquid crystalline systems for starvation, chemodynamic, and photothermal therapy of cancer
Source: Bioeng Transl Med. 2022 Dec 15;8(5):e10470. doi: 10.1002/btm2.10470 (PMC10487320; doi:10.1002/btm2.10470)
Supplement: Supplementary file 1 — APPENDIX S1. Supporting information [file BTM2-8-e10470-s001.docx]

**Supporting Information**

**Nanorod/nanodisk-integrated** **liquid crystalline systems for starvation, chemodynamic, and photothermal therapy of cancer**

Sungyun Kim^a,1^, ChaeRim Hwang^a,1^, Da In Jeong^a^, JiHye Park^a^, Han-Jun Kim^b^, KangJu Lee^c^, Junmin Lee^d^, Seung-Hwan Lee^e,f^, Hyun-Jong Cho^a,*^

^a^Department of Pharmacy, College of Pharmacy, Kangwon National University, Chuncheon, Gangwon 24341, Republic of Korea

^b^Terasaki Institute for Biomedical Innovation, Los Angeles, CA 90064, USA

^c^School of Healthcare and Biomedical Engineering, Chonnam National University, Yeosu 59626, Republic of Korea

^d^Department of Materials Science and Engineering, Pohang University of Science and Technology (POSTECH), Pohang 37673, Republic of Korea

^e^Institute of Forest Science, Kangwon National University, Chuncheon 24341, Republic of Korea

^f^Department of Forest Biomaterials Engineering, College of Forest and Environmental Sciences, Kangwon National University, Chuncheon, Gangwon 24341, Republic of Korea

^1^These authors equally contributed to this work.

^*^Corresponding author. Tel.: +82 33 250 6916; fax: +82 33 259 5631.

*E-mail address*: hjcho@kangwon.ac.kr (H.-J. Cho).


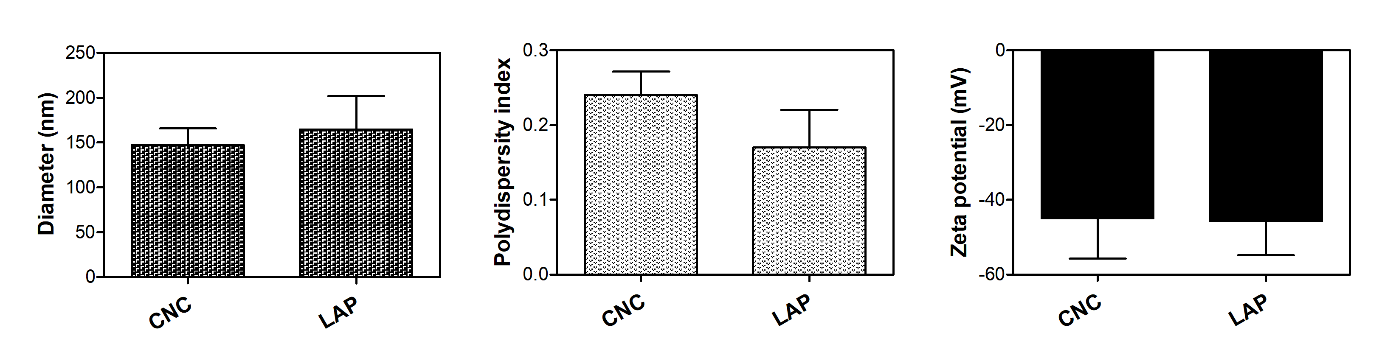


**FIGURE S1** Particle properties of CNC and LAP dispersion. Hydrodynamic diameter, polydispersity index, and zeta potential values are plotted. Each point represents mean ± SD (*n* = 3).


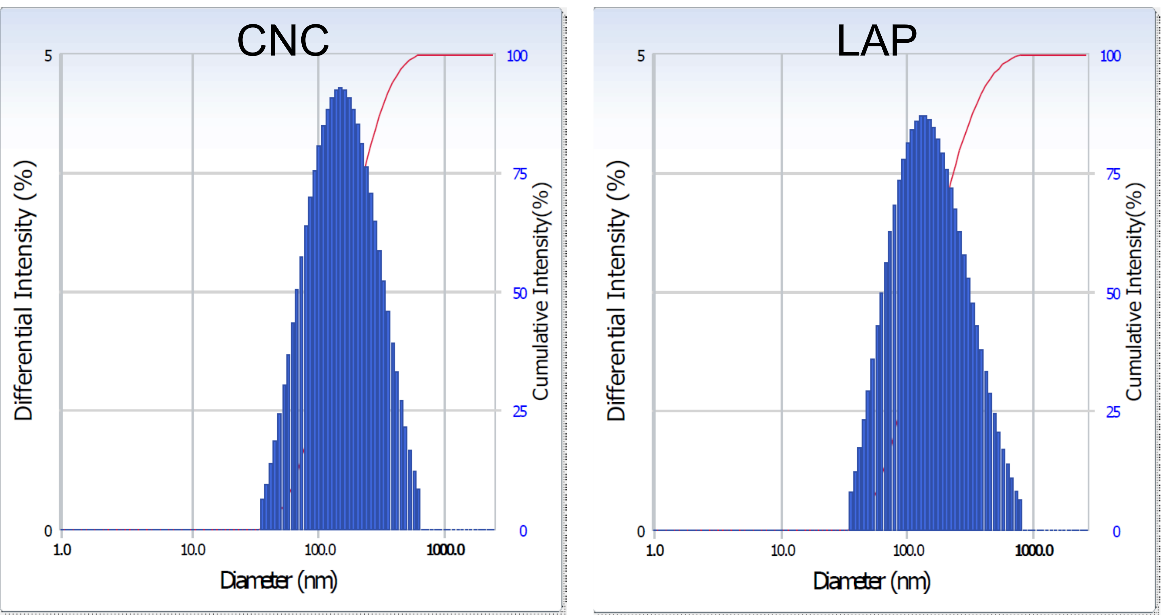


**FIGURE S2** Particle size distribution of CNC and LAP dispersion. Diameter-dependent differential intensity values are plotted.


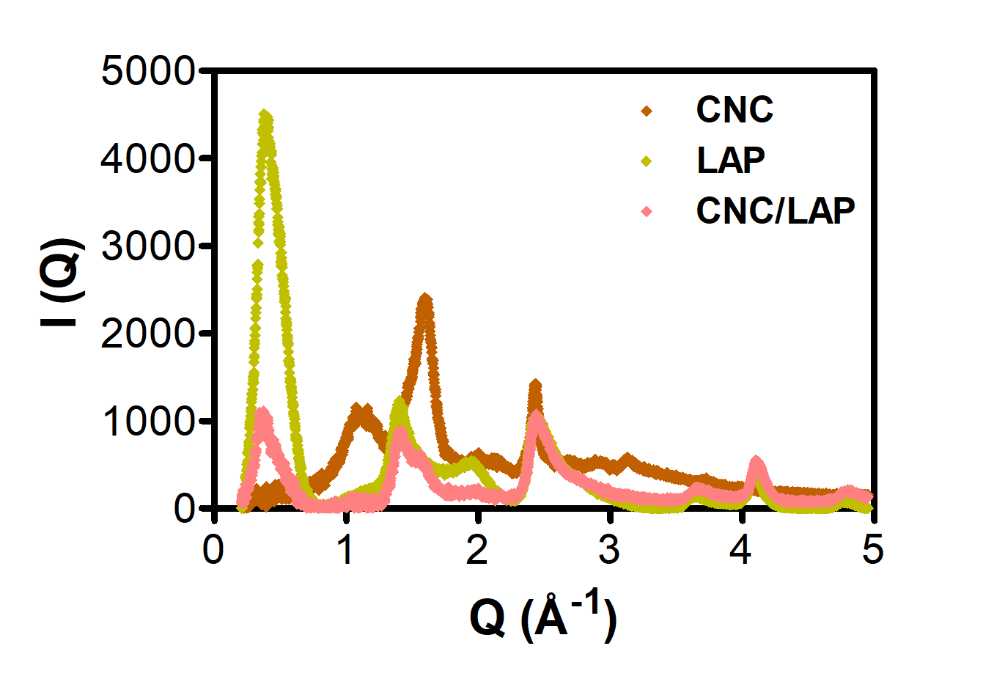


**FIGURE S3** WAXS data of CNC, LAP, and CNC/LAP.

**FIGURE S4** Polarizing microscopic images of CNC/ICG/GOx/Cu/LAP gel.





**FIGURE S5** TEM image of CNC/ICG/GOx/Cu/LAP (50/1/0.001/1/75 mg/mL, 1:50 dilution). Scale bar = 200 nm.


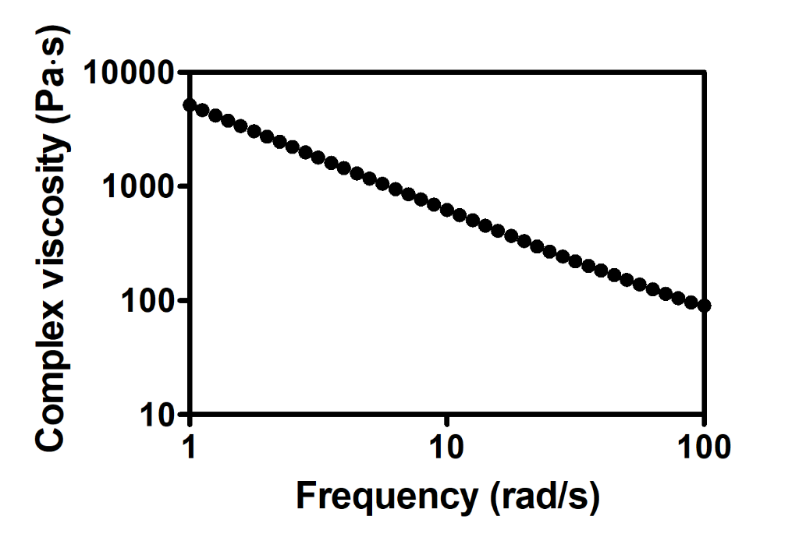


**FIGURE S6** Frequency-dependent complex viscosity profile of CNC/ICG/GOx/Cu/LAP.

**FIGURE S7** FE-SEM image of CNC/ICG/GOx/Cu/LAP gel (freeze-dried form). Scale bar = 100 μm.


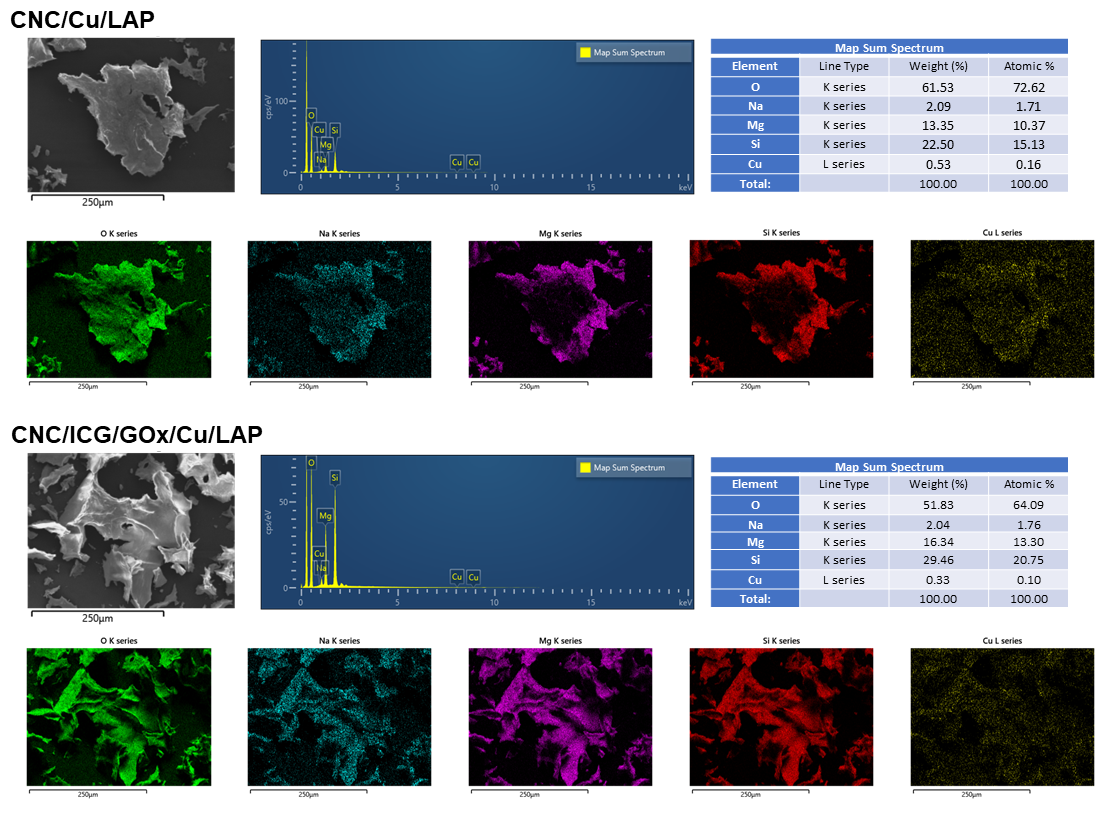


**Figure S8** EDS mapping with FE-SEM imaging for elucidating atom distribution in the lyophilized gel structure. Weight (%) and atom content (%) of O, Na, Mg, Si, and Cu in CNC/Cu/LAP and CNC/ICG/GOx/Cu/LAP groups are shown in table. Distribution images of O, Na, Mg, Si, and Cu in CNC/Cu/LAP and CNC/ICG/GOx/Cu/LAP groups are displayed. The length of scale bar is 250 μm.


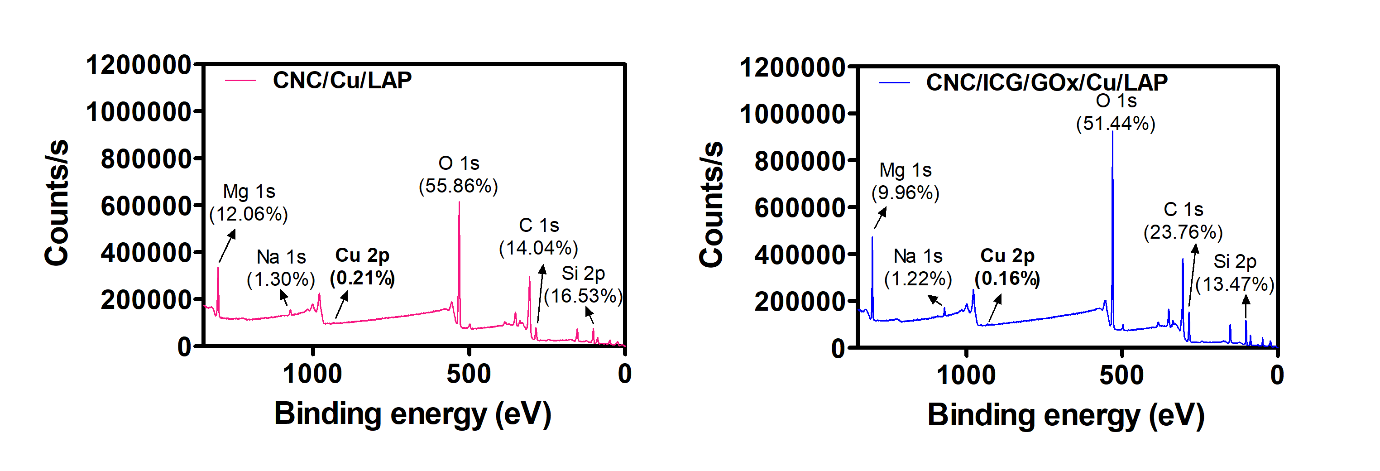


**Figure S9** XPS data of CNC/Cu/LAP and CNC/ICG/GOx/Cu/LAP groups. Atomic percentages are presented.


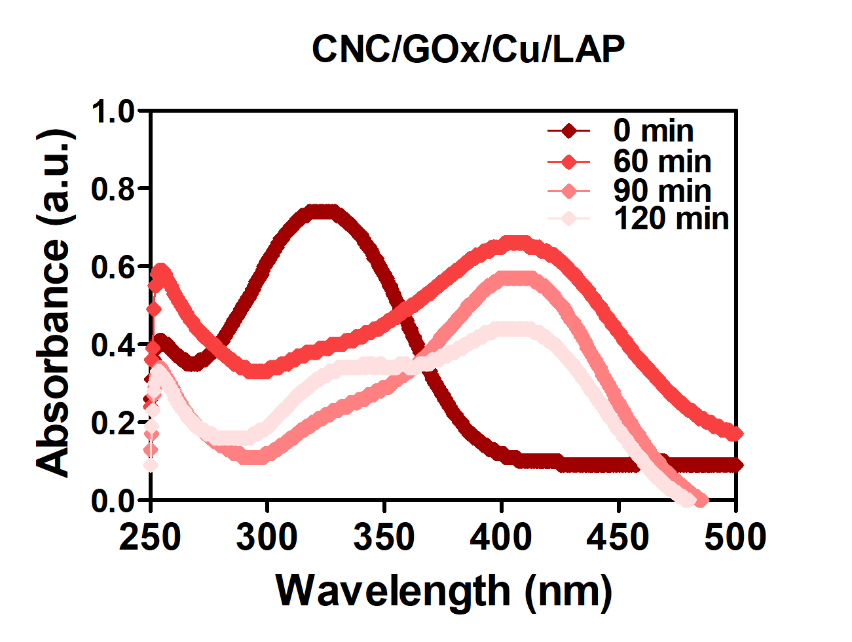


**FIGURE S10** Extracellular GSH assay according to the reaction time. Absorbance profiles of CNC/GOx/Cu/LAP group following 0, 60, 90, and 120 min reaction are plotted.


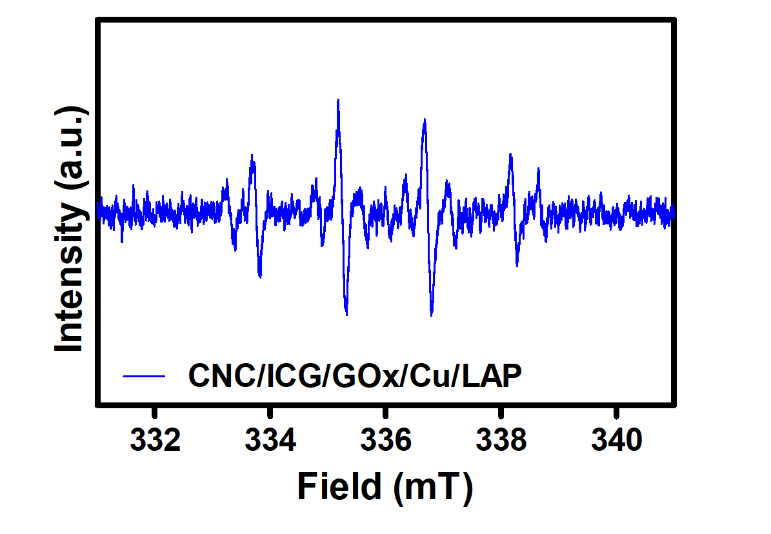


**FIGURE S11** ESR data of CNC/ICG/GOx/Cu/LAP gel.


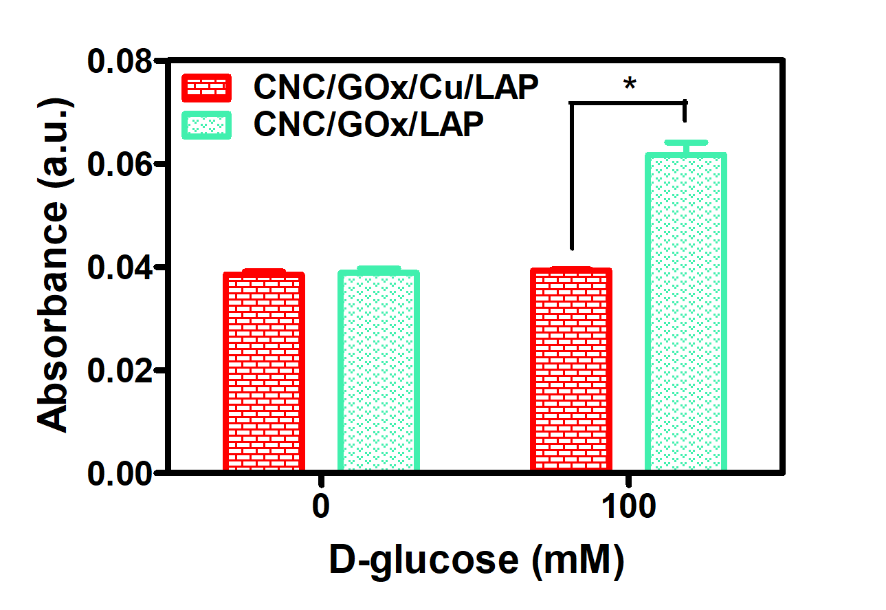


**FIGURE S12** TiOSO_4_ assay data of CNC/GOx/Cu/LAP and CNC/GOx/LAP groups. Each point represents mean ± SD (*n* = 3). ^*^*p* < 0.05, between two groups.


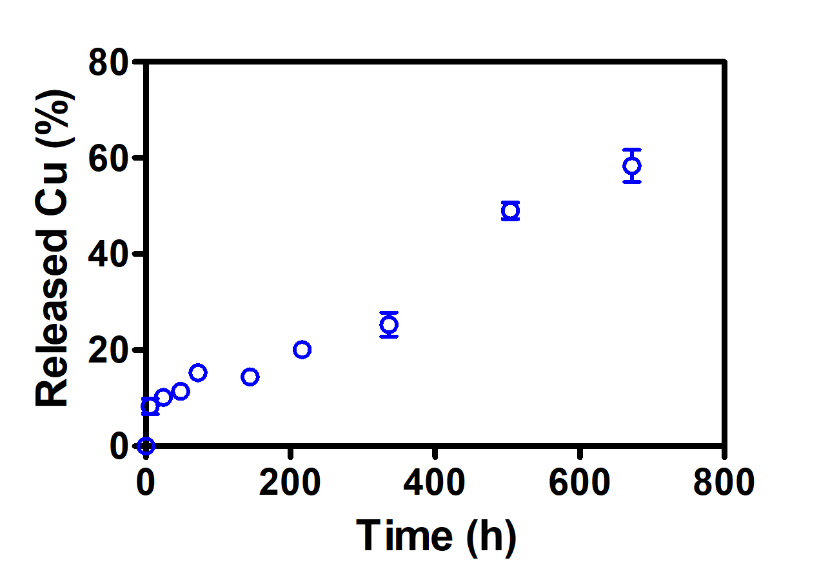


**FIGURE S13** Cu release profile from CNC/ICG/GOx/Cu/LAP gel. Each point represents mean ± SD (*n* = 3).

**FIGURE S14** Cellular uptake of ICG from CNC/ICG/GOx/Cu/LAP gel in CT-26 cells. (a) Integrated intensity profile of ICG observed by NIRF imaging. Each point represents mean ± SD (*n* = 3). (b) NIRF image of cellular accumulated ICG from CNC/ICG/GOx/Cu/LAP after 0, 10, 30, 60, 120, 360, and 1440 min of incubation.


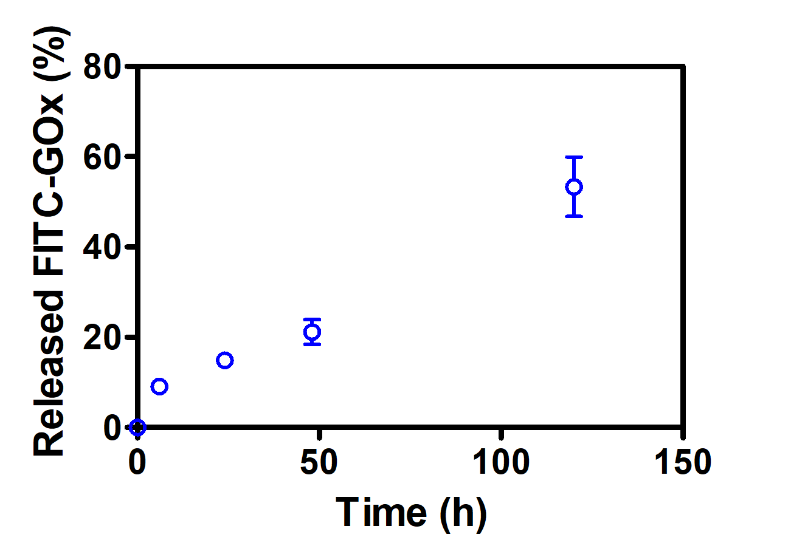


**FIGURE S15** FITC-GOx release profile from CNC/ICG/FITC-GOx/Cu/LAP gel. Each point represents mean ± SD (*n* = 3).


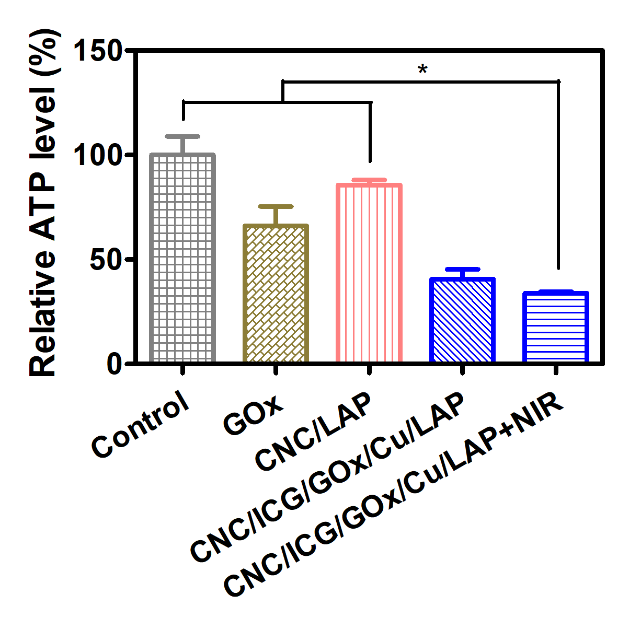


**Figure S16** ATP assay data of control, GOx, CNC/LAP, CNC/ICG/GOx/Cu/LAP, and CNC/ICG/GOx/Cu/LAP + NIR groups. Each point represents mean ± SD (*n* = 3).


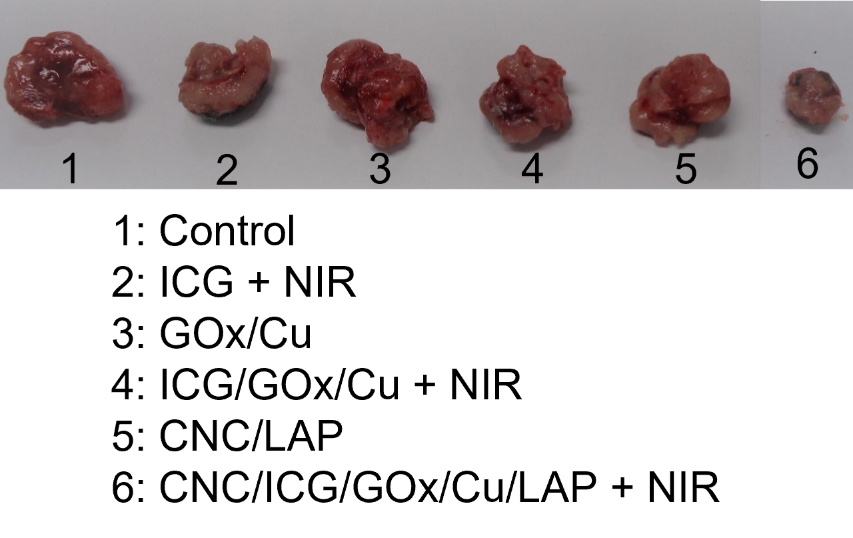


**FIGURE S17** Image of dissected tumor tissues from mouse model on final day.


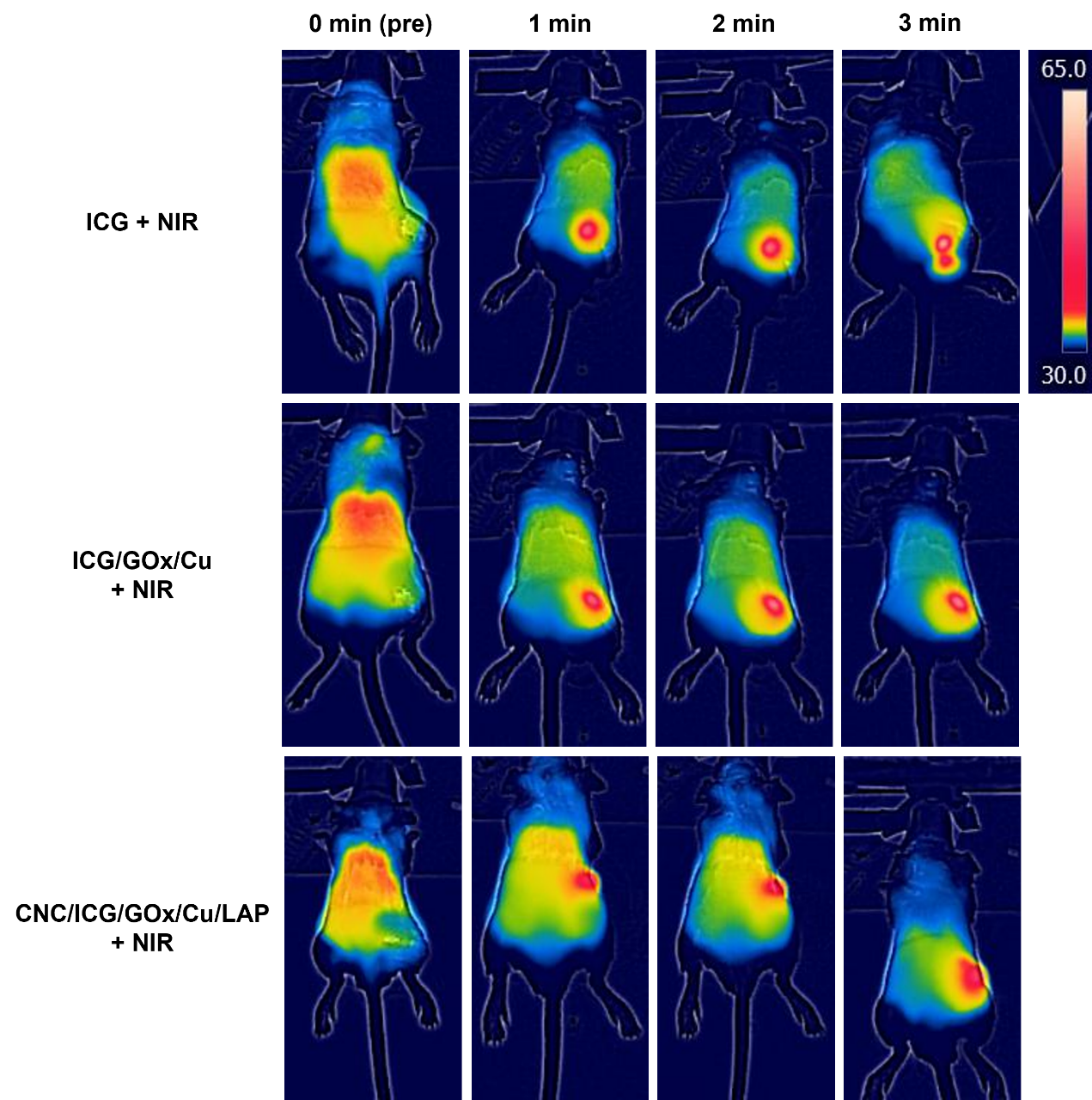


**FIGURE S18** *In vivo* thermal image of CT-26 tumor-implanted mouse on day 0 following intratumoral injection of each specimen with NIR laser exposure (for 3 min).
